# Cellular responses to halofuginone reveal a vulnerability of the GCN2 branch of the integrated stress response

**DOI:** 10.15252/embj.2021109985

**Published:** 2022-04-25

**Authors:** Aleksandra P Pitera, Maria Szaruga, Sew‐Yeu Peak‐Chew, Steven W Wingett, Anne Bertolotti

**Affiliations:** ^1^ MRC Laboratory of Molecular Biology Cambridge UK

**Keywords:** GCN2, integrated stress response, stress responses, translation, tRNA synthetase, Cancer, Pharmacology & Drug Discovery, Translation & Protein Quality

## Abstract

Halofuginone (HF) is a phase 2 clinical compound that inhibits the glutamyl‐prolyl‐tRNA synthetase (EPRS) thereby inducing the integrated stress response (ISR). Here, we report that halofuginone indeed triggers the predicted canonical ISR adaptations, consisting of attenuation of protein synthesis and gene expression reprogramming. However, the former is surprisingly atypical and occurs to a similar magnitude in wild‐type cells, cells lacking GCN2 and those incapable of phosphorylating eIF2α. Proline supplementation rescues the observed HF‐induced changes indicating that they result from inhibition of EPRS. The failure of the GCN2‐to‐eIF2α pathway to elicit a measurable protective attenuation of translation initiation allows translation elongation defects to prevail upon HF treatment. Exploiting this vulnerability of the ISR, we show that cancer cells with increased proline dependency are more sensitive to halofuginone. This work reveals that the consequences of EPRS inhibition are more complex than anticipated and provides novel insights into ISR signaling, as well as a molecular framework to guide the targeted development of halofuginone as a therapeutic.

## Graphical abstract

Pharmacological glutamyl‐prolyl‐tRNA synthetase inhibition invokes both atypical ISR‐independent translation attenuation and ISR‐mediated changes in gene expression.

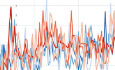

## Introduction

Halofuginone (HF), a derivative of the natural product febrifugine extracted from the hydrangea *Dichroa febrifuga,* has been used for centuries in Chinese medicine to treat malaria (Pines & Spector, 2015). Halofuginone was synthesized to alleviate the toxicity of febrifugine and has been widely utilized in veterinary practice for more than two decades to treat parasites in poultry and cattle (Daugschies *et al*, 1998). HF also exhibits a wide range of experimental and clinical activities, including antifibrotic properties, inhibition of angiogenesis and metastasis (Pines & Spector, 2015). HF binds and inhibits the EPRS with low nanomolar potency and as a result induces the ISR by activating GCN2 (Sundrud *et al*, 2009; Keller *et al*, 2012). However, recent data revealed some non‐ISR activities associated with anti‐inflammatory properties (Kim *et al*, 2020).

The ISR is a vital homeostatic pathway elicited by phosphorylation of the translation initiation factor eIF2α on serine 51 to allow cells to adapt and survive changes in their environment. ISR signaling triggers a coordinated response consisting of attenuation of translation initiation and reprogramming gene expression (Sonenberg & Hinnebusch, 2009; Wek, 2018). In humans, this response is orchestrated by four eIF2α kinases that sense different signals and two phosphatases. The kinase GCN2 is activated by amino acid shortage, PKR by double‐stranded RNA during viral infections, HRI by heme deficiency and PERK (or PEK) by accumulation of misfolded proteins in the endoplasmic reticulum (Sonenberg & Hinnebusch, 2009; Wek, 2018). Two eIF2α phosphatases reverse the activity of the four eIF2α kinases. They are split enzymes composed of a common catalytic subunit, protein phosphatase 1 (PP1), bound to one of two specific substrate receptors: stress‐inducible PPP1R15A (R15A), or the constitutive PPP1R15B (R15B) (Bertolotti, 2018). Unlike PP1 in isolation, the holoenzymes R15A‐PP1 and R15B‐PP1 are selective for their substrate (Harding *et al*, 2009; Carrara *et al*, 2017; Bertolotti, 2018). The antagonistic actions of the four eIF2α kinases and the two phosphatases tune the phosphorylation levels of eIF2α to the cellular needs and conditions.

Because of its central role in controlling cell and organismal survival, the ISR has emerged as a prime target for new pharmacological manipulations (Costa‐Mattioli & Walter, 2020; Luh & Bertolotti, 2020), particularly for approaches aimed at restoring proteostasis (Balch *et al*, 2008). Compounds that either prolong (Guanabenz (Tsaytler *et al*, 2011), Sephin1 (Das *et al*, 2015), Raphin1 (Krzyzosiak *et al*, 2018)), or block (PERK inhibitors (Atkins *et al*, 2013), ISRIB (Sidrauski *et al*, 2013)) eIF2α phosphorylation or its downstream signaling have been identified (Costa‐Mattioli & Walter, 2020; Luh & Bertolotti, 2020). Guanabenz is an approved drug, initially developed as an α2‐adrenergic agonist to treat hypertension (Holmes *et al*, 1983). It has recently shown efficacy in a phase 2 clinical trial in amyotrophic lateral sclerosis (Bella *et al*, 2021), 10 years after its activity as a proteostatic compound was revealed (Tsaytler *et al*, 2011). Like Guanabenz, Sephin1, a non‐adrenergic guanabenz derivative (Tsaytler *et al*, 2011; Das *et al*, 2015; Krzyzosiak *et al*, 2018), as well as Raphin1, prolong eIF2α phosphorylation and protect cells and mice from protein misfolding stress and associated diseases (Luh & Bertolotti, 2020). Sephin1 has passed through a favorable phase 1 clinical trial (https://clinicaltrials.gov/ct2/show/NCT03610334). The development of PERK inhibitors has stopped due to on‐target pancreatic toxicity (Atkins *et al*, 2013). ISRIB has shown benefit in mouse models of diverse diseases (Costa‐Mattioli & Walter, 2020) and the development of its derivatives is evaluated for vanishing white matter disease (Wong *et al*, 2019). Because HF has progressed to phase 2 clinical trials for the treatment of HIV‐Related Kaposi's Sarcoma and Duchenne muscular dystrophy treatment (www.clinicaltrials.gov), it is a potentially attractive compound to explore the benefit of ISR modulation both experimentally and clinically.

Here, we dissected the mechanism of action of HF and revealed that although HF induces the two canonical ISR adaptations consisting of attenuation of bulk protein synthesis and gene expression reprogramming, this response is surprisingly atypical because translation attenuation occurs independently of GCN2 and eIF2α phosphorylation. We found that these changes following HF treatment are all rescued upon proline addition, demonstrating that they result from EPRS inhibition. The knowledge that the observed activities of HF are all on‐target provides the molecular basis to select specific disease conditions for optimal responsiveness to the compound, as exemplified here with the increased sensitivity of proline‐dependent cancer cells to HF treatment.

## Results

### Atypical ISR induction by HF

**Figure 1:**
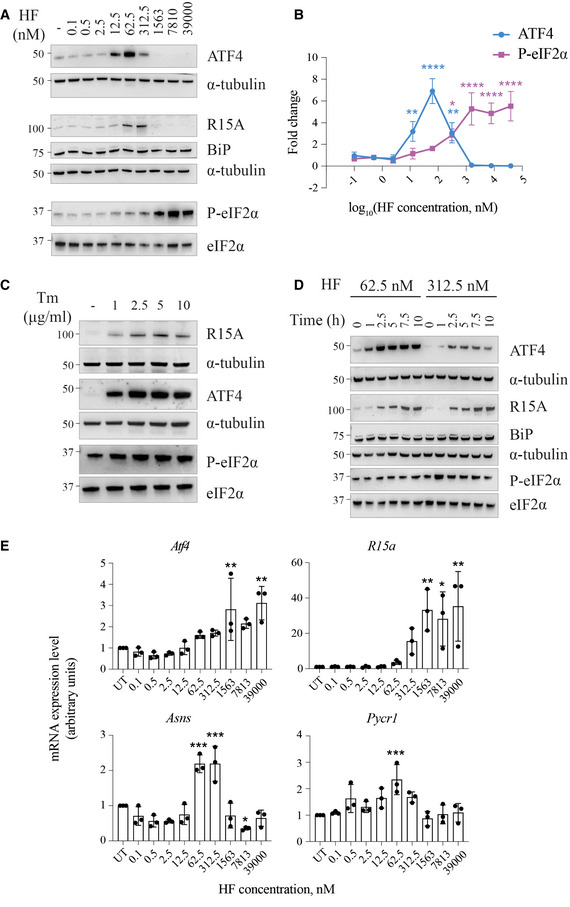
HF induces an atypical blunted ISR Representative immunoblots of the indicated proteins in lysates from HeLa cells treated with indicated concentrations of halofuginone (HF) for 5 h.Quantification of ATF4 and P‐eIF2α from experiments as in (A). Data are mean ± SD (*n* = 3 biological replicates). *P ≤ 0.0414, **P ≤ 0.0019, ****P < 0.0001, as determined by one‐way ANOVA with Dunnett’s multiple comparison test.Similar to (A), but with HeLa cells treated with indicated concentrations of tunicamycin for 5 h.Similar to (A), but using lysates from HeLa cells treated with 62.5 nM and 312.5 nM HF for indicated times.Relative abundance of the indicated mRNAs detected by qPCR in lysates from HeLa cells treated with indicated concentrations of HF for 5 h. Data are mean ± SD (*n* = 3 biological replicates). *P ≤ 0.0388, **P ≤ 0.0046, ***P ≤ 0.0008, as determined by one‐way ANOVA with Dunnett’s multiple comparison test. Data information: Representative results of at least three independent experiments are shown in each panel.

**Figure EV1:**
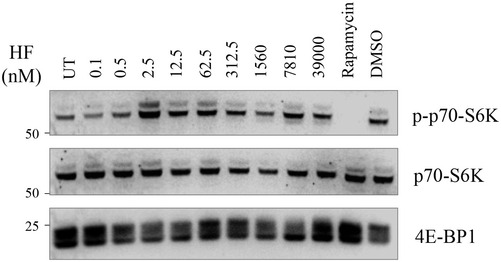
mTORC1 activation upon HF treatment Representative immunoblots of indicated proteins in lysates from HeLa cells treated for 5 h with indicated concentrations of HF or 200 nM Rapamycin for 3 h.

To comprehensively characterize the activities of HF, we started by conducting a dose‐response treatment of HeLa cells and monitored induction of key ISR markers. As anticipated (Sundrud *et al*, 2009; Keller *et al*, 2012; Misra *et al*, 2021), HF induced an ISR response manifested by increased phosphorylation of eIF2α and increased levels of ATF4, R15A (Fig 1A and B). Surprisingly, ATF4 and R15A were induced from 12.5 nM to 312.5 nM but no longer with higher concentrations of HF. Under these conditions, eIF2α phosphorylation continued to increase (Fig 1A and B) either because of the loss of R15A expression or because of persistent kinase activation or both. In contrast to HF, tunicamycin, which activates the PERK branch of the ISR, induced phosphorylation of eIF2α and increased ATF4 and R15A levels in a dose‐dependent manner (Fig 1C). The blunted ISR at high concentrations of HF was also present in other studies (Sundrud *et al*, 2009; Keller *et al*, 2012; Misra *et al*, 2021), but the mechanism underlying this phenomenon remains unknown, a knowledge gap which motivated this investigation. We next examined whether the lack of induction of ATF4 and R15A seen at concentrations of HF above 312.5 nM was a complete loss or a kinetic delay. Thus, we conducted time course experiments at 62.5 nM, a concentration of HF leading to typical ISR induction, as well as 312.5 nM, a concentration where ISR induction was dampened (Fig 1A). Prolonged treatment with 312.5 nM HF did not rescue the high induction of ATF4 observed at 62.5 nM (Fig 1D). As previously reported (Misra *et al*, 2021), mTORC1 signaling was elevated following HF, as manifested by an increased phosphorylation of the ribosomal S6 kinase (Fig EV1). This is because mTORC1 is activated due to an increased abundance of amino acids (Misra *et al*, 2021), possibly as consequence of ISR induction.

We next monitored changes in abundance of ISR mRNA targets (Wek, 2018). As previously observed with other ISR inducers (Dey *et al*, 2010; Schneider *et al*, 2020; Misra *et al*, 2021), HF caused a dose‐dependent increase of *Atf4* and *R15a* transcripts (Fig 1E). Expression of asparagine synthetase (ASNS) is controlled transcriptionally by ATF4 (Wek, 2018). HF increased accumulation of *Asns* transcripts at 62.5 and 312.5 nM (Fig 1E) but this did not occur at higher concentrations (Fig 1E). A similar pattern was observed for *Pycr1,* another ATF4 target (Nilsson *et al*, 2014) encoding a proline biosynthetic enzyme (Fig 1E). Thus, the dose‐response of *Asns* and *Pycr1* mRNA to HF precisely mirrored that of its transcriptional factor ATF4, peaking at 62.5 nM (Fig 1A and E). These detailed time course and dose‐response analyses reveal that HF induces an atypical ISR, as this response is blunted downstream of eIF2α phosphorylation at high concentrations of the compound.

### ISR‐dependent HF activities

**Figure 2:**
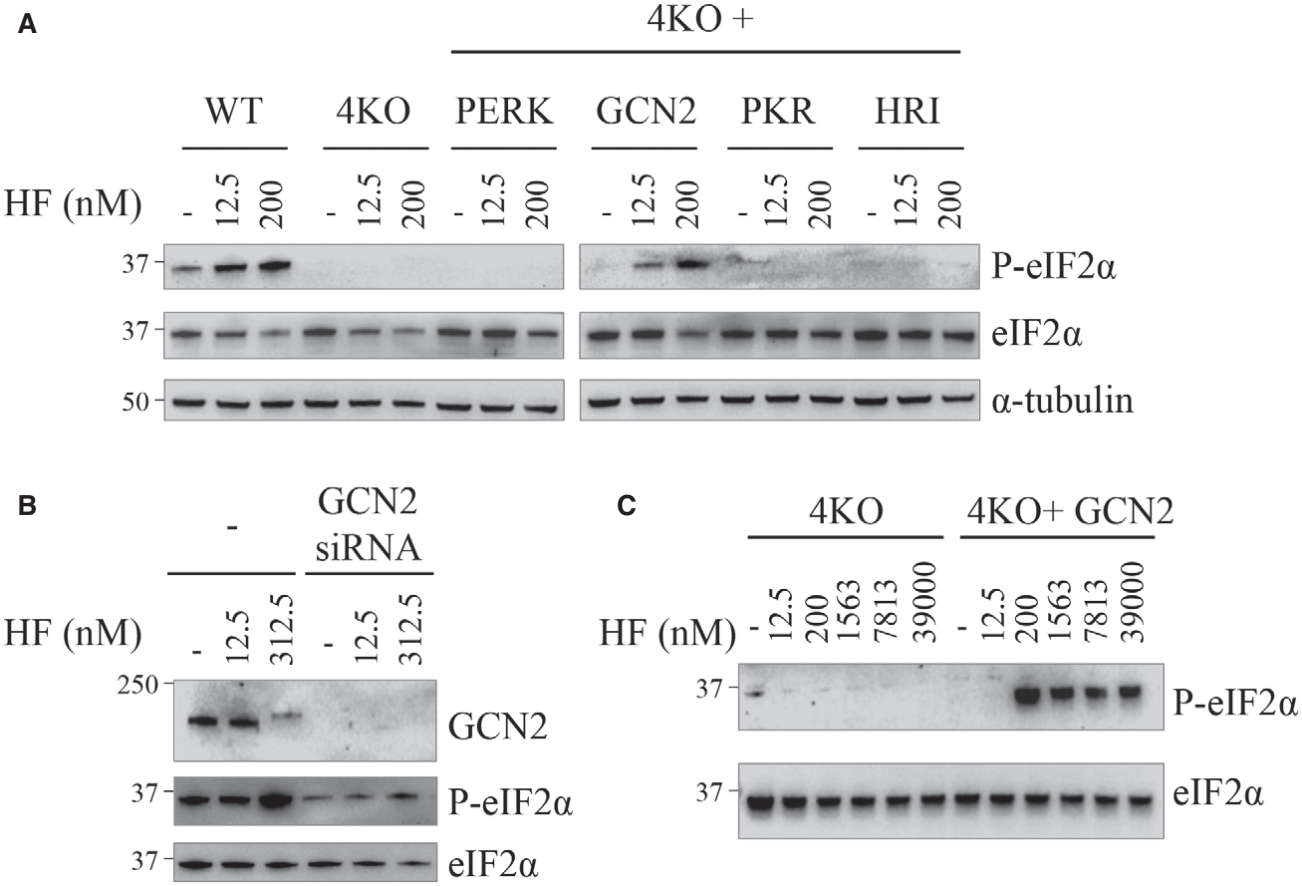
GCN2 mediates eIF2α phosphorylation by HF Representative immunoblots of indicated proteins in lysates from mouse embryonic fibroblasts (MEFs) of indicated genotype after treatment with 12.5 nM or 200 nM HF for 5 h.Similar to (A), but using HeLa cells untreated or treated with GCN2 siRNA 48 h before treatment with indicated concentrations of HF for 5 h.Similar to (A), but with high HF concentrations. Data information: Representative results of at least three independent experiments are shown in each panel.

Because HF was recently reported to display some ISR‐independent activities (Kim *et al*, 2020), we next examined if the activities observed here were ISR‐related or off‐target. eIF2α phosphorylation in response to various stresses is completely abolished in cells lacking all four eIF2α kinases (4KO; Taniuchi *et al*, 2016). However, signal‐specific induction of eIF2α phosphorylation is restored upon reintroduction of the cognate kinase in the 4KO cells (Taniuchi *et al*, 2016). This provides a robust single eIF2α kinase system to examine ISR sensing. We monitored induction of eIF2α phosphorylation by HF in wild‐type mouse embryonic fibroblasts (MEFs), 4KO cells, and in 4KO cells complemented by each of the four human eIF2α kinases (Taniuchi *et al*, 2016). HF induced eIF2α phosphorylation in WT cells but not in the 4KO cells and this was rescued upon complementation with GCN2 in the 4KO cells, but no other eIF2α kinases (Fig 2A). This demonstrates that eIF2α phosphorylation by HF is entirely dependent on GCN2 in mouse cells. To validate these findings, we knocked down GCN2 in HeLa cells (Fig 2B). siRNA targeting *Gcn2* effectively eliminated GCN2 and as a consequence, cells were unable to increase eIF2α phosphorylation upon HF treatment (Fig 2B). In the 4KO cells treated up to 39 μM of HF, there was no detectable increase in eIF2α phosphorylation, in contrast to the 4KO cells complemented by GCN2 (Fig 2C). These results reveal that induction of eIF2α phosphorylation by HF is entirely dependent on GCN2.

**Figure 3:**
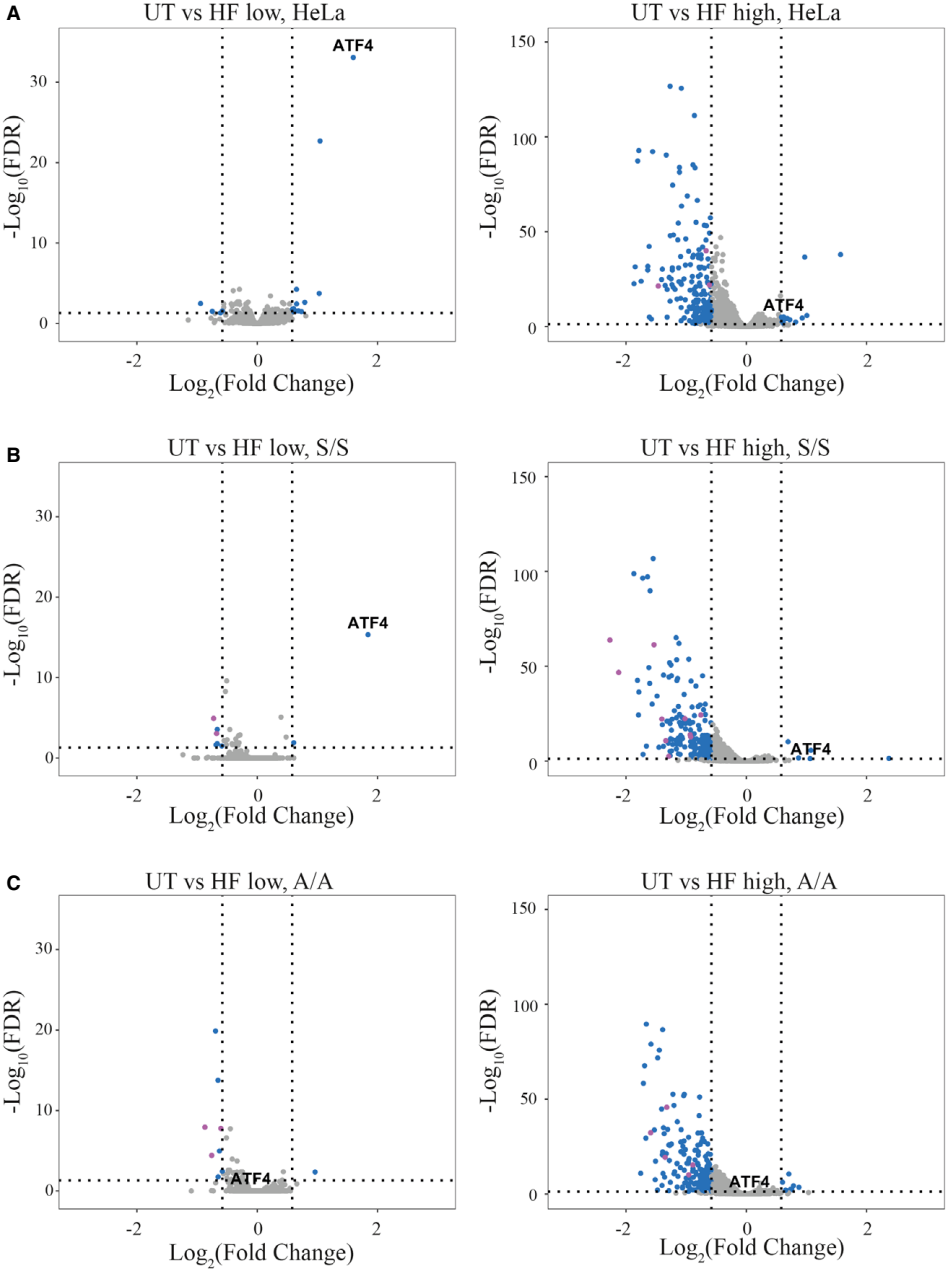
Global quantitative proteomic analyses of cell response to HF Tandem mass tag mass spectrometry analyses performed in triplicate from HeLa cells treated with 12.5 or 312.5 nM HF for 5 h.Similar to (A), but using eIF2α^S/S^ MEF cells treated with 12.5 or 200 nM HF for 5 h.Similar to (B), but using eIF2α^A/A^ cells. Data information: Results are presented as pairwise comparisons. Data points in magenta represent collagen proteins. Vertical dashed lines indicate Log_2_(Fold change) = 0.58 and that corresponds to 1.5‐fold change.

We next used quantitative proteomics with tandem mass tag mass spectrometry as an unbiased approach to investigate the molecular basis for the unusual disconnect between high eIF2α phosphorylation and ATF4 induction at high HF concentrations. Discrete changes were observed in HeLa cells treated with 12.5 nM of HF for 5 h, with only 12 proteins found increased by the treatment (*P* ≤ 0.05, fold change ≥ 1.5), ATF4 showing the highest induction (Fig 3A and Dataset EV1). This validated the approach and demonstrated the selectivity of HF as an ISR inducer. Induction of ATF4 increased 3‐fold after 5 h treatment with 12.5 nM of HF, and only 1.5‐fold with 312.5 nM HF in these quantitative proteomic analyses performed in HeLa cells (Fig 3A). ATF4 also increased in HF‐treated wild‐type mouse embryonic fibroblasts (eIF2α^S/S^) but not in eIF2α^A/A^ cells that are incapable of phosphorylating eIF2α (Scheuner *et al*, 2001; Fig 3B and C). As in HeLa cells, ATF4 induction was lower in eIF2α^S/S^ cells treated with 200 nM HF than in cells treated with 12.5 nM (Fig 3B). This demonstrates that HF induces ATF4 through the canonical GCN2‐to‐eIF2α signaling pathway but this induction is dampened at high concentrations of HF.

Whilst only a few proteins increased in abundance upon treatment of HeLa or MEF cells with 12.5 nM HF, 312.5 nM HF decreased the levels of 188 proteins (*P* ≤ 0.05, fold change ≥ 1.5) in HeLa, 204 in eIF2α^S/S^ and 195 in eIF2α^A/A^ cells (Fig 3 and Dataset EV1). Gene ontology analyses were performed (Dataset EV2). Amongst the different categories, enrichments for genes involved in collagen fibril organization, including various types of collagens were observed (Dataset EV1). Decreased collagen expression could provide the molecular basis for HF antifibrotic activities (Pines & Spector, 2015). This suggested that some of the medically relevant activities of HF may be a consequence of the decreased abundance of downregulated proteins. We then searched for the underlying mechanism.

### High concentrations of HF decrease R15B

**Figure 4:**
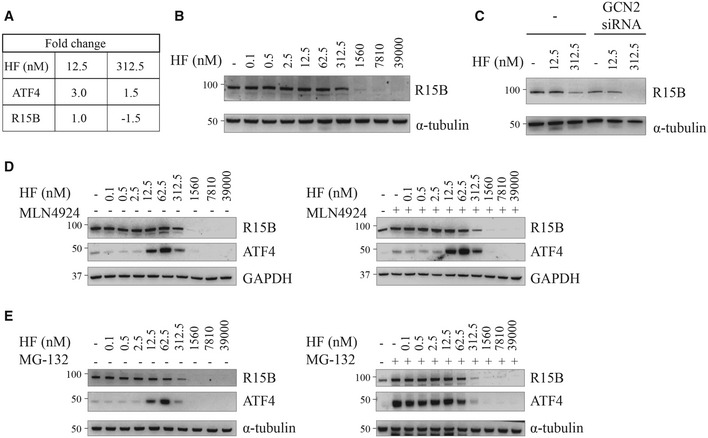
HF decreases R15B abundance independently of GCN2 and protein degradation Fold change of ATF4 and R15B after 5 h treatment with 12.5 or 312.5 nM HF relative to untreated cells. Data obtained from the quantitative proteomic analyses shown in Fig 3A.Representative immunoblots of indicated proteins in lysates from HeLa cells after 5 h treatment with indicated concentrations of HF.Similar to (B), but using HeLa cells untreated or treated with GCN2 siRNA 48 h before treatment with indicated concentrations of HF.Representative immunoblots of lysates from HeLa cells treated with indicated concentrations of HF for 5 h with or without the Nedd8‐activating enzyme inhibitor MLN4924 (1 μM).Representative immunoblots of lysates from HeLa cells treated with indicated concentrations of HF for 5 h with or without proteasome inhibitor MG‐132 (10 μM). Data information: Representative results of at least three independent experiments are shown in each panel.

**Figure EV2:**
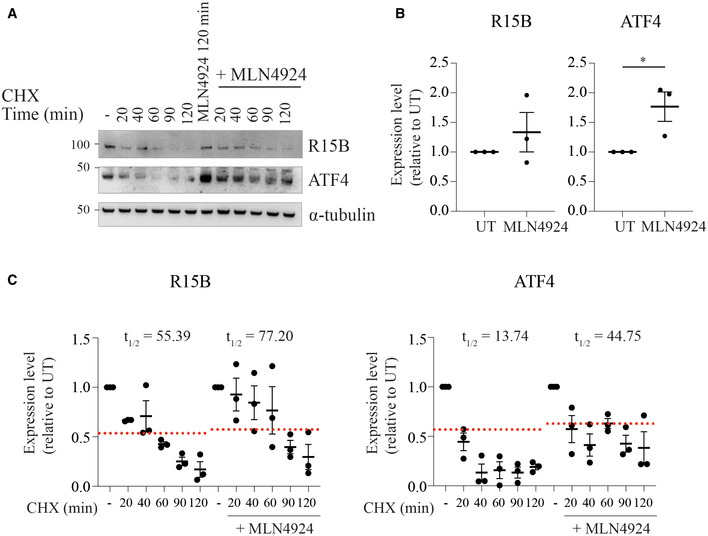
The Nedd8‐activating enzyme inhibitor MLN4924 stabilizes ATF4 and R15B Representative immunoblots of the indicated proteins in lysates from HeLa cells treated with cycloheximide (20 μg/ml) with or without the inhibitor of the NEDD8‐activating enzyme MLN4924 (1 μM) for indicated times.Quantification of R15B and ATF4 in cells treated with MLN4924 (1 μM) for 120 min. Data are mean ± SEM (*n* = 3 biological replicates). *P = 0.0369, as determined by unpaired *t*‐test.Quantification of experiments such as the ones shown in (A). t_1/2_ values were calculated with results from 3 independent experiments. Data are mean ± SEM (*n* = 3 biological replicates).

Amongst the proteins downregulated at high HF concentration was R15B (Fig 4A). This was unexpected because R15B is resistant to the translation attenuation resulting from eIF2α phosphorylation (Andreev *et al*, 2015; Schneider *et al*, 2020). Because the decreased abundance of R15B (Fig 4A) could explain the unusually high increase in eIF2α phosphorylation observed at high HF concentrations (Fig 1A), we investigated it further. First, we confirmed that R15B was detectable up to 312.5 nM HF (Fig 4B). However, in cells treated for 5 h at higher concentrations than 312.5 nM HF, R15B was essentially depleted (Fig 4B). Surprisingly, this decrease was GCN2‐independent and even exacerbated in GCN2‐depleted cells (Fig 4C). Thus, we next examined whether the depletion of R15B observed at high concentrations of HF resulted from an increased degradation. As previously reported, R15B (Jousse *et al*, 2003) and ATF4 (Rutkowski *et al*, 2006) are unstable proteins. Both R15B and ATF4 are targeted for degradation by the cullin‐RING E3 ligase β‐TRCP (Lassot *et al*, 2001; Coyaud *et al*, 2015). The activity of such ligases is controlled by NEDD8‐activating enzyme, which can be inhibited by the small‐molecule inhibitor MLN4924 (Soucy *et al*, 2009). Both R15B and ATF4 proteins were stabilized in cells treated with MLN4924 (Fig EV2A–C), confirming that they are targeted for degradation by a cullin‐RING ligase. The decreased abundance of ATF4 and R15B in cells treated with high concentrations of HF was not affected by treatment with MLN4924 (Fig 4D) indicating that it does not result from increased degradation. To confirm these observations, we treated cells with the proteasome inhibitor MG‐132. Proteasome inhibition resulted in a marked accumulation of ATF4 and a slight increase in R15B in absence of HF (Fig 4E). However, MG‐132 did not prevent the loss of ATF4 and R15B observed after treatment with high HF concentrations, above 312.5 nM (Fig 4E). Thus, the loss of ATF4 and R15B at high HF concentrations does not result from increased degradation.

### Translational changes upon HF treatment

**Figure 5:**
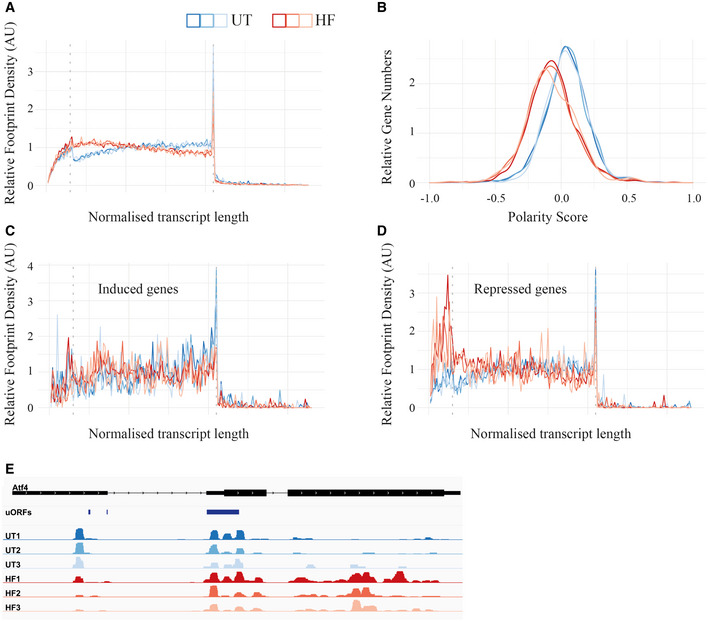
Global analyses of translational changes upon HF treatment Metagene analysis plot showing average ribosome occupancy from all genes aligned at start codons in MEF cells untreated (UT, 3 replicates in blue) or treated with 100 nM HF (3 replicates in red) for 6 h. The vertical dashed lines, from left to right, separate the length‐normalized transcripts into the (i) 5′UTR, (ii) CDS and (iii) 3′UTR. All genes were included (8,712–7,889 transcripts depending on the sample).Polarity scores distribution for all genes. Negative values correspond to footprint enrichment at the 5′ end of a CDS.Metagene analysis plot for genes identified with increased translation efficiency following HF treatment (180–167 transcripts).Similar to (C), but for genes with repressed translational efficiency following HF treatment. (335–293 transcripts).Plot representing ribosome density across the *Atf4* mRNA in untreated cells (UT1‐3, in blue) and treated with HF (HF1‐3, in red). Black lines represent introns, intermediate‐sized blocks denote UTRs, and thick blocks represent CDS. uORF1 and uORF2, split across an intron, are represented. Note that the 6 tracks representing ribosome density on *Atf4* mRNA have been scaled such that the maximum value in each track has a fixed height. Data information: 5A–E were analyzed from (Misra *et al*, 2021).

To gain further mechanistic insights into HF response, we next focused on translation. We first took advantage of a published ribosome profiling dataset (Misra *et al*, 2021) and performed metagene analyses to characterize the translational response to HF at a global level. The distribution of ribosomes on mRNAs at a genome‐wide level in control cells appeared as expected (Vorontsov *et al*, 2021), with a gradient of footprint density toward the 3’ end attesting high translation activity (Fig 5A). A low ribosome density after stop codons revealed efficient translation termination in all conditions (Fig 5A). HF treatment caused a striking genome‐wide redistribution of ribosomes, with an increased density at the beginning of the ORFs and a decreased density toward the 3’ end (Fig 5A). This difference was confirmed by a polarity score analysis (Fig 5B) and may be explained by the fact that HF causes ribosome pausing at proline codons (Misra *et al*, 2021), as this would result in fewer ribosomes reaching the end of the transcript. Following treatment with HF, translation of some mRNAs decreased, others increased, whilst the majority remained unchanged (Misra *et al*, 2021). We next performed metagene analyses of ribosome footprints in the differentially regulated groups of transcripts. The increased 5’ end occupancy on the ORFs observed at the global level after HF was not seen on transcripts that were preferentially translated upon HF (Fig 5C). The translationally repressed mRNAs displayed an increased ribosome density in their 5’ UTR (Fig 5D). This has been seen at a genome‐wide levels in yeast exposed to amino acid starvation (Ingolia *et al*, 2009; Schuller *et al*, 2017) and is consistent with the notion that uORF translation in 5’ UTR often represses translation of the main ORFs (Hinnebusch *et al*, 2016). We then focused on *Atf4* translation. High ribosome occupancy was observed on the uORFs but not on the main ORF of *Atf4* in untreated cells (Fig 5E). HF (100 nM) increased occupancy of ribosomes on the main ORF (Fig 5E), in agreement with the increased expression of the protein observed here (Fig 1A).

From these diverse analyses, the genome‐wide shift of ribosome density toward the 5’ end of coding regions observed at a global level is the most intriguing because it is reminiscent to the redistribution caused in yeast upon depletion of a translation elongation factor (Schuller *et al*, 2017). This suggests that HF causes an elongation defect. This was unexpected because the GCN2‐dependent eIF2α phosphorylation observed upon HF treatment (Figs 1 and 2) is expected to cause attenuation of translation initiation.

### HF decreases translation in a GCN2‐ and eIF2α‐independent manner

**Figure 6:**
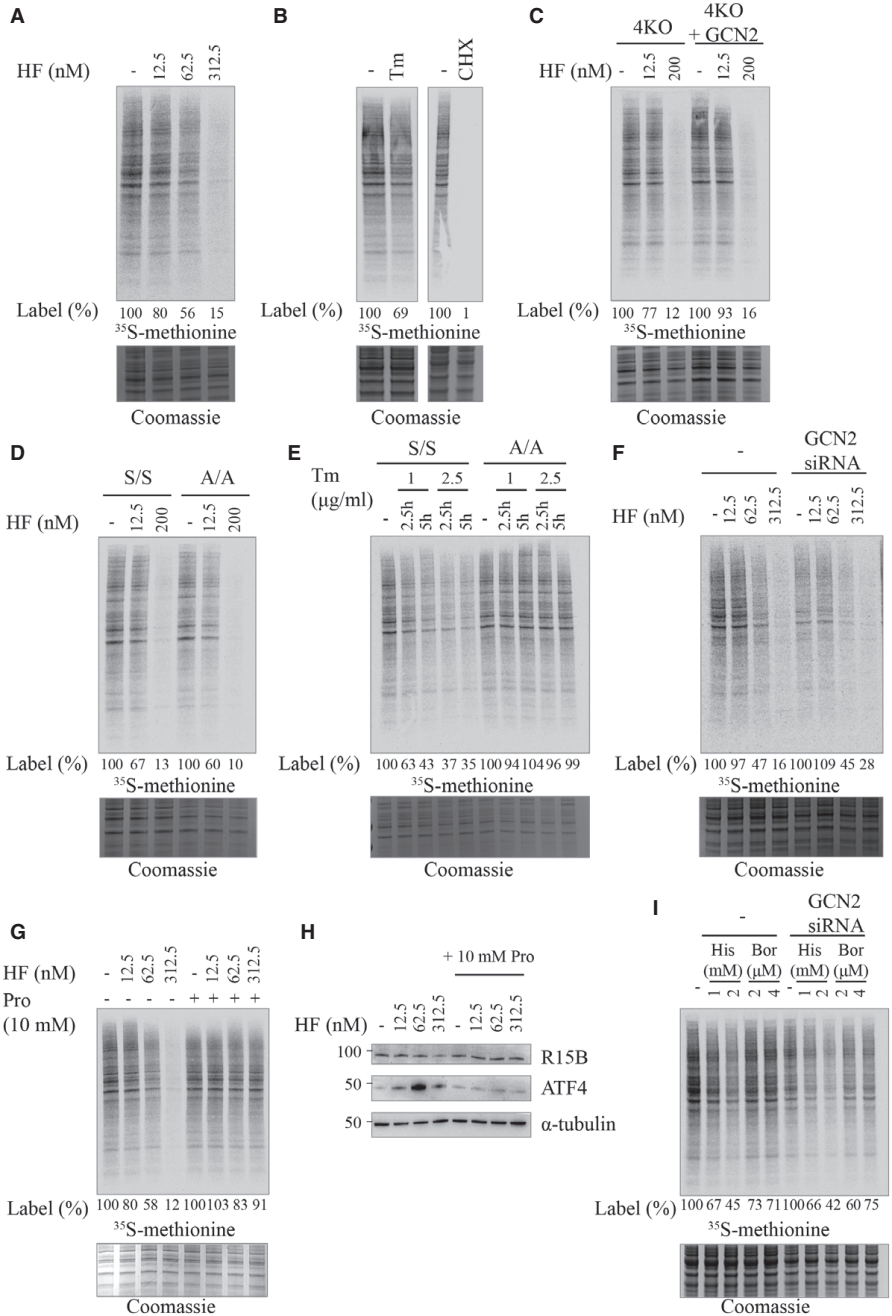
Translation attenuation in response to tRNA synthetase inhibitors is independent of the ISR Newly synthesized proteins pulse‐labeled with ^35^S‐methionine for 10 min in HeLa cells pre‐treated with indicated compounds for 2.5 h, except (E) (Bottom) Coomassie‐stained gel.Newly synthesized proteins in HeLa cells treated with 2.5 μg/ml Tm or 50 μg/ml cycloheximide (CHX).Newly synthesized proteins in 4KO or 4KO+GCN2 MEF cells treated with 12.5 or 200 nM HF.Newly synthesized proteins in eIF2α^S/S^ and eIF2α^A/A^ MEF cells treated with 12.5 or 200 nM HF.Newly synthesized proteins in eIF2α^S/S^ and eIF2α^A/A^ MEF cells treated with indicated concentrations of Tm for 2.5 or 5 h.Newly synthesized proteins in HeLa cells untreated or treated with GCN2 siRNA 48 h before treatment with indicated concentrations of HF.Newly synthesized proteins in HeLa cells treated with indicated concentrations of HF with or without proline supplementation (10 mM).Representative immunoblots of indicated proteins in lysates from HeLa cells after treatment with indicated concentrations of HF with or without proline supplementation (10 mM).Newly synthesized proteins in HeLa cells untreated or treated with GCN2 siRNA 48 h before treatment with indicated concentrations of histidinol (His) or borrelidin (Bor). Data information: Quantification of experiments such as the ones shown in (C), (D) and (I) are presented in Figs EV3 and EV4.

**Figure EV3:**
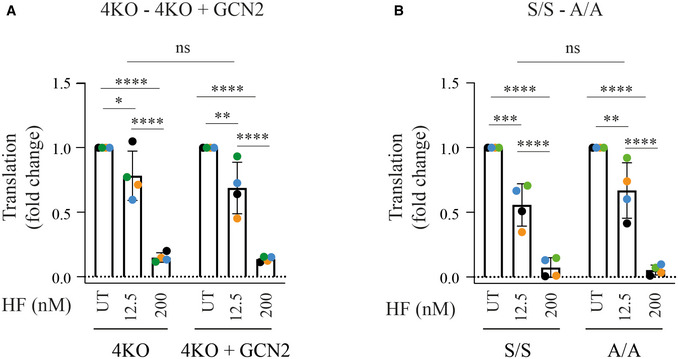
Translational attenuation following HF is independent of GCN2 or eIF2α phosphorylation A, BQuantification of ^35^S‐methionine labeling in experiments such as the ones shown in Fig 6C and D, respectively. Data are mean ± SD (*n* ≥ 3 biological replicates). *P ≤ 0.0376, **P ≤ 0.0031, ***P ≤ 0.0001, ****P < 0.0001, ns: not significant, as determined by two‐way ANOVA.

We next measured translation in cells treated with various concentrations of HF. Treatment of HeLa cells with 12.5, 62.5, and 312.5 nM HF decreased translation by ~20, 40, and 85%, respectively (Fig 6A). For comparison, the ISR‐dependent translation attenuation induced by tunicamycin was ~30%, in contrast to the general translation inhibitor cycloheximide that blocked translation completely (Fig 6B). This suggested that the 40 and 85% decrease in translation at high HF concentrations may be ISR‐independent. Thus, we next analyzed the consequence of HF treatment in the 4KO cells completely defective in ISR sensing. Surprisingly, a dose‐dependent translation attenuation was detected in the 4KO cells at 12.5 and 200 nM HF (Fig 6C). Quantification of four replicates revealed that translation attenuation in the 4KO or 4KO+GCN2 cells was not statistically significant (Figs 6C and EV3). This was unexpected. Thus, we performed similar experiments in eIF2α^A/A^ cells. The dose‐dependent decrease in translation upon HF treatment was not significantly different in eIF2α^S/S^ and eIF2α^A/A^ cells (Figs 6D and EV3). In contrast, translation attenuation induced by tunicamycin was completely abolished in the eIF2α^A/A^ cells (Fig 6E), as expected (Scheuner *et al*, 2001). This confirms our ability to detect ISR‐dependent translational changes and reveals that the translation attenuation upon HF treatment is atypically ISR‐independent in MEFs, requiring neither GCN2 nor eIF2α phosphorylation. This conclusion was corroborated in human cells, with translation attenuation upon HF treatment being resistant to GCN2 knockdown (Fig 6F).

HF is a competitive inhibitor of the EPRS and as such, its inhibitory activity can be rescued by proline (Keller *et al*, 2012). Addition of 10 mM proline rescued translation and prevented induction of ATF4 even at high concentrations of HF (Fig 6G and H). Importantly, proline supplementation also prevented the decrease in R15B observed at high HF concentrations (Fig 6H). This reveals that although the translation attenuation resulting from HF treatment occurs independently of the ISR, it is an on‐target effect due to inhibition of prolyl‐tRNA synthetase.

**Figure EV4:**
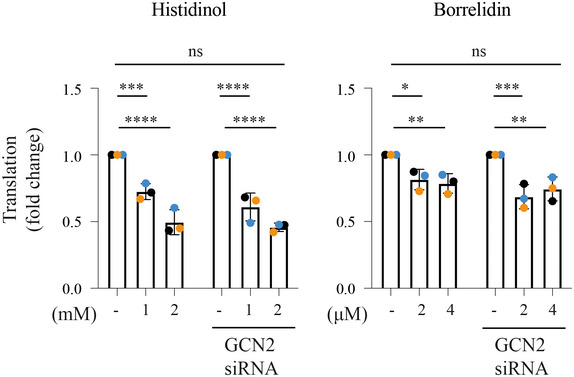
Translational attenuation following histidinol or borrelidin is independent of GCN2 Quantification of ^35^S‐methionine labeling in experiments such as the ones shown in Fig 6I. Data are mean ± SD (*n* = 3 biological replicates). *P ≤ 0.0116, **P ≤ 0.0044, ***P ≤ 0.0004, ****P < 0.0001, ns: not significant, as determined by two‐way ANOVA.

We next tested two other compounds, histidinol (Hansen *et al*, 1972) and borrelidin (Francklyn & Mullen, 2019), impairing histidine and threonine tRNA charging, respectively. Both compounds attenuated translation from ~25 to 55% in wild‐type cells (Fig 6I). Similarly to HF, translation attenuation occurred to a similar magnitude in cells where GCN2 had been knocked down (Figs 6I and EV4). This suggests that the observations made using HF might have broad relevance to diverse conditions perturbing tRNA charging.

### Vulnerability of proline‐dependent cancer cells to HF

**Figure 7:**
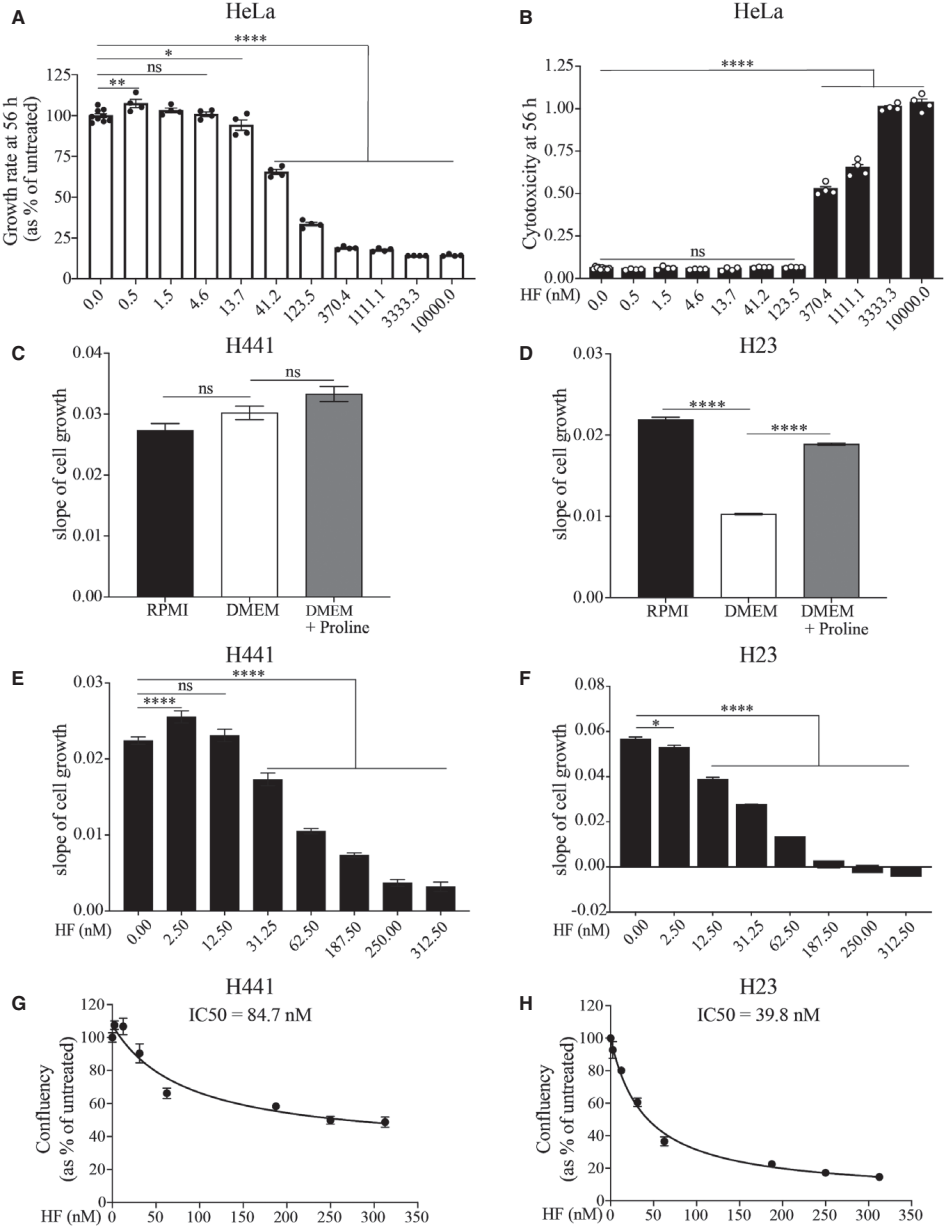
Proline‐dependent cells are sensitized to the cytotoxic effects of HF A, BGrowth rate and cytotoxicity (CellTox Green Dye) at 56 h of HeLa cells treated with indicated concentrations of HF and monitored at 37°C in an Incucyte S3 system (Fig EV5A). Data are mean (*n* ≥ 4 biological replicates) ± SEM. *P = 0.0481, **P = 0.0047, ****P < 0.0001, as determined by one‐way ANOVA with Dunnett’s multiple comparison test. ns: not significant.C, DSlope of cell growth of H441 (C) and H23 (D) lung cancer cell lines grown for 72 h in RPMI (containing 20 mg/l Proline) media versus DMEM (Proline‐free) or DMEM supplemented with 20 mg/l Proline. Data are mean (*n* = 4 biological replicates) ± SEM. ****P < 0.001 as determined by one‐way ANOVA with Dunnett’s multiple comparison test. ns: not significant. Slope of cell growth was determined by simple linear regression curve fit of cellular growth rate in distinct conditions over 72 h presented in Fig EV5C and D.E, FSlope of cell growth of H441 (E) and H23 (F) cells exposed to indicated concentrations of HF for 72 h at 37°C. Data are mean (*n* = 3 biological replicates) ± SD. *P = 0.0151, ****P < 0.0001, as determined by one‐way ANOVA with Dunnett’s multiple comparison test. Slope of cell growth was determined by simple linear regression curve fit of cellular growth rate in distinct conditions over 72 h presented in Fig EV5E and F.G, HAnalysis of IC50 values of HF on confluency at 72 h endpoint of H441 (G) and H23 (H) cells normalized to control conditions and determined by nonlinear regression curve fit for inhibitory dose‐response (IC50). Each point represents mean (*n* = 3 biological replicates) ± SD.

**Figure EV5:**
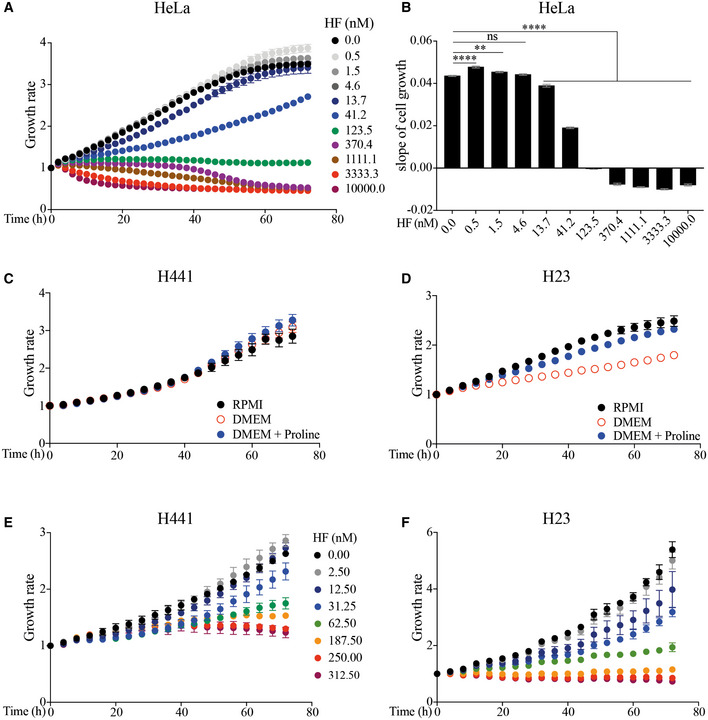
Differential effect of HF on growth rates of HeLa, H441 and H23 cells A, BGrowth rates (A) and the corresponding slopes of cell growth (B) of HeLa cells treated with indicated concentrations of HF and monitored for 72 h at 37°C in an Incucyte S3 system. Slope of cell growth was determined by simple linear regression curve fit of exponential cellular growth rate (0–56 h) in distinct conditions. Data are mean (*n* ≥ 4 biological replicates) ± SEM. **P = 0.0073, ****P < 0.0001, as determined by one‐way ANOVA with Dunnett’s multiple comparison test. ns: not significant.C, DGrowth rates of H441 (C) and H23 (D) lung cancer cell lines grown for 72 h at 37°C in an Incucyte S3 system in RPMI (containing 20 mg/l Proline) media versus DMEM (Proline‐free) or DMEM supplemented with 20 mg/l Proline. Data are mean (*n* = 4 biological replicates) ± SEM.E, FGrowth rates of H441 (E) and H23 (F) cells exposed to indicated concentrations of HF for 72 h at 37°C in an Incucyte S3 system. Data are mean (*n* = 3 biological replicates) ± SD.

We next measured the vulnerability of HeLa cells exposed to HF. HF did not substantially impair cell growth and caused no measurable cytotoxicity in HeLa cells at 56 h post treatment up to 13 nM (Figs 7A and B, and EV5A and B), a concentration that induced the ISR (Fig 1A) and decreased translation by ~ 20–30% (Fig 6). Above 13 nM, HF had cytostatic effects (Fig 7A). HF manifested cytotoxic effects from 370 nM (Fig 7B), a concentration causing an 85% decrease in translation (Fig 6A). This suggests a threshold in the tolerability to translation attenuation.

Some cancer cells are dependent on proline for proliferation (Loayza‐Puch *et al*, 2016) and decreased proline biosynthesis impairs their growth (Sahu *et al*, 2016), a property that might be harnessed therapeutically using HF. As a proof of concept, we selected two lung adenocarcinoma cell lines, NCI‐H441 and NCI‐H23, with different proline dependency (Sahu *et al*, 2016) and assessed if they presented different vulnerability to HF. First, we used a previously described experimental paradigm and tested cell growth in DMEM, a media lacking non‐essential amino acids (NEAAs), amongst which proline, and RPMI, a media containing NEAAs (Fig EV5C and D). Growth of NCI‐H441 was not impaired in DMEM whilst NCI‐H23 cells proliferated more in RPMI (Fig 7C and D). Addition of proline rescued the growth of NCI‐H23 in DMEM (Fig 7D). We performed a dose‐response of HF in these two cell lines (Fig EV5E and F) and observed that NCI‐H23 cells were more sensitive to HF than NCI‐H441 cells (Fig 7E–H). This provides a proof of concept to target cancer types presenting an increased dependency on proline for treatment with HF.

## Discussion

Through a comprehensive analysis of the mechanism of action of HF, a tRNA synthetase inhibitor that has progressed to phase 2 clinical trials for various indications, our work reveals a vulnerability of the GCN2 branch of the ISR.

In contrast to tunicamycin‐induced and PERK‐mediated translation attenuation which is entirely driven by the ISR, we found that the decrease in protein synthesis following HF treatment is ISR‐independent because it is not significantly different in wild‐type cells, GCN2 knockout or eIF2α^A/A^ MEFs and in human cells where GCN2 has been knocked down. However, the gene expression reprogramming following HF, which is robust up to a threshold in wild‐type cells, is completely abolished in cells lacking either GCN2 or a phosphorylable allele of eIF2α. At high concentrations of HF, the expression of ISR‐inducible targets such as ATF4 and R15A is lost because translation is completely blocked upon inhibition of EPRS, explaining why the ISR is blunted under these conditions.

The ISR‐dependent and independent changes following HF treatment are both rescued upon proline addition, revealing that they result from an on‐target effect as a consequence of EPRS inhibition. Similar to amino acid deprivation, EPRS inhibition by HF results in accumulation of uncharged tRNA (Misra *et al*, 2021). Interestingly, arginine deprivation has been reported to decrease translation partly through GCN2 and partly through ribosome pausing (Darnell *et al*, 2018). Ribosome pausing also occurs upon HF treatment (Misra *et al*, 2021). Both events, accumulation of uncharged tRNA and colliding ribosomes activate GCN2 (Wek *et al*, 1989; Dong *et al*, 2000). Analyses of ribosome profiling following HF treatment reveals a change in polarity in ribosome density with an increased 5’ end occupancy on ORFs. This profile is similar to the redistribution of ribosomes observed in yeast upon depletion of a translation elongation factor (Schuller *et al*, 2017), advocating that HF caused an elongation defect as a consequence of ribosome pausing. Whilst activated GCN2 usually phosphorylates eIF2α to reduce translation initiation and protect cells form various insults (Wek, 2018), here, we find that activation of GCN2‐to‐eIF2α signaling upon HF treatment is not robust enough to pre‐empt from translational elongation defects. The findings reported here using HF reveal a vulnerability of the GCN2 branch of the ISR and may have relevance to the response to amino acid limitation and tRNA synthetase deficiencies in mammals. Supporting this possibility, we found that histidinol and borrelidin, impairing histidine and threonine charging, respectively, also cause translation attenuation with no measurable contribution of GCN2.

In contrast, the attenuation of protein synthesis evoked by tunicamycin is entirely mediated by the ISR, as it is virtually abolished in eIF2α^A/A^ cells. The distinct molecular profiles of the tunicamycin‐induced PERK signaling and HF‐induced GCN2 signaling reveals some important divergence of these two ISR arms. GCN2 is the only eIF2α kinase in yeast whilst mammals have evolved PERK, PKR, and HRI to sense different stimuli. Yeast cells lacking GCN2 are viable but manifest decreased resistance to amino acid deprivation (Dever *et al*, 1992). In human, mutations in *EIF2AK4*, encoding GCN2, cause a loss of function associated with pulmonary veno‐occlusive disease (PVOD), a rare form of pulmonary arterial hypertension (Montani *et al*, 2016). PVOD has both a genetic and an environmental component and develops in individuals lacking a functional GCN2 upon exposure to drugs, toxins, or radiation (Montani *et al*, 2016). Thus, loss of GCN2 in human is viable and, in most cases, not detrimental. In contrast, loss of function mutations in *EIF2AK3* encoding PERK cause an inevitably fatal autosomal recessive disorder characterized by permanent neonatal or early infancy insulin‐dependent diabetes (Delépine *et al*, 2000). Genetic inactivation of *EIF2AK3 or EIF2AK4* in mice recapitulates the human features. Mice lacking GCN2 are viable, fertile and develop normally under standard conditions (Zhang *et al*, 2002), in contrast to mice lacking PERK, which die shortly after birth due to failure to maintain glucose homeostasis (Harding *et al*, 2001). Although not essential for healthy life under most conditions, the GCN2 pathway can be protective in mammals, with the liver of *EIF2AK4* knockout mice being sensitized to HF (Misra *et al*, 2021). Our findings suggest that this phenotype may not be contributed by differences in translation rates but by the other aspects of GCN2 signaling, such as ATF4 induction.

Attenuation of translation initiation mediated by tunicamycin via PERK is robust contrasting that resulting from GCN2 upon HF. This finding may provide a molecular rationale to explain why, in mammals, lack of GCN2 is viable in contrast to the lack of PERK. GCN2 is the sensor of an intracellular pathway that maintains amino acid homeostasis and is critical to maintain cell fitness in yeast (Dever *et al*, 1992). In mammals, amino acid homeostasis is largely maintained through interorgan transport (Brosnan, 2003) suggesting that the reliance on the GCN2 pathway might have decreased from unicellular to metazoans. This shift from intracellular to interorgan amino acid homeostasis from unicellular to metazoan could explain why GCN2 does not measurably contribute to translation attenuation in response to HF. In contrast, PERK, which appeared more recently in evolution, is essential in humans and attenuation of translation initiation via PERK is robust.

Cancer cells are highly proliferative and therefore have increased requirements for amino acids that need to be met with exogenous supplies and upregulation of *de novo* synthesis of non‐essential amino acids (Efeyan *et al*, 2015; Mossmann *et al*, 2018). Such cancer‐specific alterations can be exploited therapeutically. Indeed, L‐asparaginase depletes asparagine and slows tumor growth, a property that has been exploited clinically for the treatment of acute lymphoblastic leukemia and non‐Hodgkin lymphomas (Richards & Kilberg, 2006). Emerging yet robust and diverse evidence indicates that the metabolism of proline, a non‐essential amino acid, is altered in numerous cancers (D’Aniello *et al*, 2020). A gene expression study across nearly 2000 tumors identified genes encoding the proline biosynthesis enzymes, PYCR1 and ALDH18A1, amongst the most upregulated ones (Nilsson *et al*, 2014). Importantly, both PYCR1 and ALDH18A1 are ATF4 target genes (D’Aniello *et al*, 2015). A separate ribosome profiling study corroborated these findings revealing a shortage of proline resulting in paused ribosomes at proline codons in diverse cancer types, a deficit associated with high expression of PYCR1 (Loayza‐Puch *et al*, 2016). The benefit of targeting proline metabolism to reduce cancer growth has been evaluated experimentally with a large body of evidence showing that genetic inactivation of PYCR1 reduces growth of diverse cancer types (Loayza‐Puch *et al*, 2016; Elia *et al*, 2017; D’Aniello *et al*, 2020). The model emerging from these studies suggests that some cancer cells have an increased proline requirement, which they try to meet by upregulating proline biosynthetic enzymes. Thus, proline limitation could be a therapeutic paradigm applicable to these various types of cancers. Here, we provide proof‐of‐concept data to achieve this using HF. Because HF has already gone through phase 2 clinical trials, the idea of using HF to target proline‐dependent cancer types can be directly tested.

In addition to the role of proline in supporting cell growth, it also constitutes 25% of the amino acids in collagen, the main protein component of the extracellular matrix (D’Aniello *et al*, 2020). Importantly, we find that collagens are amongst the top downregulated targets upon HF treatment in cells in culture, and this is manifested at nanomolar doses of the drug. The decreased synthesis of collagen also explains the antifibrotic properties of HF (Pines & Spector, 2015).

This study rationalizes diverse activities of HF in one unifying mechanism and reveals that the consequences of EPRS inhibition are surprisingly more complex than anticipated. The data presented here shed light on unusual aspects of the ISR and provide a molecular framework to consider treating proline‐dependent tumors with HF. Considering the large number of cancer types with increased proline requirements, developing HF for cancer treatments, alone or in combination with other drugs, can have a broad range of benefits.

## Materials and Methods

### Compounds

Halofuginone (#32481), Tunicamycin (#T7765), Histidinol (#6647), Borrelidin (#B3061), and Cycloheximide (#C7698) were purchased from Sigma‐Aldrich, MLN4924 (#S7109‐SEL) was purchased from Stratech and MG‐132 (#1748) was purchased from Tocris Bioscience.

### Cell culture

HeLa cells were cultured in Dulbecco’s Modified Eagle’s Media (DMEM, #D5796, ThermoFisher Scientific) supplemented with penicillin, streptomycin (#15140122, ThermoFisherScientific), glutamine (#25030081, ThermoFisherScientific), and 10% fetal bovine serum (FBS, #10270106, ThermoFisher Scientific). *eIF2α* ^S/S^, *eIF2α* ^A/A^ and 4KO (+/‐ kinase) MEF cells were cultured in DMEM supplemented with penicillin, and glutamine, 55 μM β‐mercaptoethanol (#M3148, Sigma‐Aldrich), 1X non‐essential amino acids (#11140‐050, ThermoFisher Scientific) and 10% FBS.

NCI‐H441(ATCC^®^ HTB‐174™) and NCI‐H23 (ATCC^®^ CRL‐5800™) cell lines (both early passages from ATCC) were cultured in RPMI 1640 Medium (ATCC modification, #11504566, ThermoFisher Scientific) supplemented with penicillin, streptomycin, glutamine, and 10% FBS.

All mammalian cell lines were grown in a humidified incubator with 5% CO_2_ at 37°C.

For GCN2 knockdown experiments, calculated volumes of Opti‐MEM (#11058021, ThermoFisher Scientific), ON‐TARGETplus Human GCN2 siRNA (#L‐005314‐00‐0005, Horizon Discovery Biosciences Limited) and Lipofectamine RNAiMAX (#13778‐150, ThermoFisher Scientific) were mixed together and then pipetted to the wells (200 μl/well in 12‐well plate). After 10‐min incubation, cells resuspended in DMEM were added on top of the transfection mix. Treatment with HF was performed 48 h after transfection and media was exchanged to fresh DMEM one day preceding the treatment.

### Assessment of cell growth and viability

For proline dependency and HF sensitivity studies, cells were plated as follows: HeLa cells (50,000 cells/ml) in 96‐well plates (0.1 ml/well) in DMEM media, H441 cells (80,000 cells/ml) in 24‐well plates (0.5 ml/well) in RPMI media, and H23 cells (30,000 cells/ml) in 24‐well plates (0.5 ml/well) in RPMI media. In HF sensitivity experiments, the plating density of the H441 versus H23 cells was set to result in similar confluency at the start of the treatment. Cells were incubated for 24 h at 37°C before starting the treatments. To test proline dependency, the media of H441 and H23 cells was exchanged to either control RPMI media, DMEM or DMEM supplemented with 20 mg/l proline. To assess HF sensitivity, cells were treated with increasing concentrations of HF by adding 2× concentrated HF in culture media to the cells. For HeLa cells, a 1:2,000 dilution of the CellTox Green Dye (#G8731, Promega) was added to the media to assess cytotoxicity by normalizing the Green Dye confluency to Phase Confluency signal. For both proline dependency and HF sensitivity, the proliferation and viability of the cells was recorded every 2–4 h for 72 h post treatment with the Incucyte S3 Live‐Cell Analysis System (Sartorius, Essen BioScience) with a 10× objective. Growth rates of cells in distinct conditions were determined by correcting the recorded cellular confluency over time to the measured plating density at time 0 h (start of the treatment).

### Western blot and antibodies

HeLa or MEF cells (90,000 cells/ml) were plated in 12‐well plates (1 ml/well) the day before treatment. The time course was performed in reverse chronological order to allow synchronous cell collection. At the end of treatments, cells were washed with PBS and lysed in 150 μl Laemmli Buffer. Lysates were boiled at 95°C for 5 min, sonicated and resolved on Bolt SDS‐PAGE 4–12% Bis‐Tris gels or 3–8% Tris‐Acetate gels (ThermoFisher Scientific). Proteins were transferred to nitrocellulose membranes using the Trans‐Blot Turbo Transfer System (Bio‐Rad). Membranes were blocked in 5% skimmed milk and then probed with primary antibodies followed by incubation with the appropriate horseradish peroxidase‐conjugated secondary antibodies (Promega). Proteins were visualized using ECL Prime Western Blotting System (#RPN2232, Cytiva) and imaged in a ChemiDoc Touch system (Bio‐Rad). Bands were quantified using ImageStudioLite, and analyses were performed using GraphPad software. The primary antibodies used were as follows: ATF4 (Proteintech (#10835‐1‐AP), 1:1,000), α ‐tubulin (Sigma‐Aldrich (#T5168), 1:5,000), p‐eIF2α (Abcam (#ab32157), 1:1,000), eIF2α (Abcam (#ab26197), 1:1,000), BiP (BD Biosciences (#610978), 1:1,000), GAPDH (Millipore (#MAB374), 1:5,000), GCN2 (Cell Signaling (#3302), 1:1,000), p‐p70 S6K (Cell Signaling (#9205), 1:1,000), p70 S6K (Cell Signaling (#9202), 1:1,000), Ppp1r15a (Proteintech (#10449‐1‐AP), 1:1,000), Ppp1r15b (in‐house mouse monoclonal antibody 3E11, to be described elsewhere, 1:1,000), 4E‐BP1 (Cell Signaling (#9644), 1:1,000).

### Quantitative RT‐PCR

HeLa or MEF cells (90,000 cells/ml) were plated in 12‐well plates (1 ml/well) one day before treatment. RNA was extracted using RNeasy Mini Kit (QIAGEN) and treated with DNase (RNase‐Free DNase Set, QIAGEN) according to the manufacturer’s instructions. RNA concentration was measured using a NANODROP1000 spectrophotometer (ThermoFisher Scientific), and 0.5 μg RNA was reverse transcribed to cDNA using iScript cDNA Synthesis Kit (#1708891, Bio‐Rad). RNA levels of indicated genes were assessed in the quantitative PCR on a Viia 7 Real‐Time PCR system (Applied Biosystems) using SYBR^®^ Select Master Mix (#4472908, Applied Biosystems) and the following primers: *Atf4:* AGTTCGACTTGGATGCCCTG (F) – CCAACGTGGTCAGAAGGTCA (R)*, R15a:* TGAAGCCTGGGGACTTTTGG (F) – CCTCTAGGGACACTGGTTGC (R), *Asns:* TTGCACACAGAGGTCCAGATG (F) – CATTCCAAACAGCGGGTCAA (R), *Pycr*1: CTCCCTGCTCATCAACGCT (F) – GTCCAGCTTCACCTTGTCCA (R). RNA levels are presented relative to the house keeping gene *β‐actin*–GGGCATGGGTCAGAAGGATT(F), TCGATGGGGTACTTCAGGGT (R) and expressed as a fold change.

### Assessment of translation rates

HeLa or MEF cells (80,000 cells/ml) were plated in 24‐well plates (0.5 ml/well). The following day cells were treated as indicated. After the treatment, cells were labeled with direct addition of 100 μCi/ml ^35^S‐methionine (Hartmann Analytic) for 10 min at 37°C (150 μl fresh media containing 1.5 μl ^35^S‐methionine was added to each well). Note that cells were not cultured in methionine‐free media prior to the labeling because methionine depletion is a potent ISR inducer. The labeled cells were then washed twice with PBS and lysed in 75 μl Laemmli Buffer. Lysates were boiled at 95°C for 5 min, sonicated and resolved on Bolt SDS‐PAGE 4%–12% Bis‐Tris gels (ThermoFisher Scientific). Gels were then stained with Coomassie Blue, destained and after the gels had been imaged, they were transferred to a 20% ethanol, 7% acetic acid, and 4% glycerol solution for 10 min. The gels were transferred to filter paper and dried using a gel dryer. Gels were then exposed to a Storage Phosphor Screen (GE Healthcare) and analyzed by phosphorimaging using a Typhoon Imager Scanner (GE Healthcare).

### Proteomics

Cells were grown on 150 × 20 mm plates and were treated with indicated concentrations of HF one day after plating. After the treatment, cells were washed and scraped in ice cold PBS and spun at 4°C for 10 min at 12,000 rpm. Cell pellet was resuspended in urea buffer (8 M urea, 75 mM NaCl, 50 mM Tris pH 7.4) and then sonicated. The lysate was centrifuged at 4°C for 10 min at 12,000 rpm. Pierce BCA Protein Assay Kit (#23250, ThermoFisher Scientific) was used to measure protein concentration in the supernatant.

Protein samples in urea buffer were reduced with 5 mM DTT at 56°C for 30 min and alkylated with 10 mM iodoacetamide in the dark at room temperature for 30 min. The samples were then diluted to 4 M urea and digested with Lys‐C (Promega) for 4 h at 25°C. Next, the samples were further diluted to 1.6 M urea and digested with trypsin (Promega) over night, at 30°C. Digestion was stopped by the addition of formic acid (FA) to a final concentration of 0.5%. Any precipitates were removed by centrifugation at 13,000 rpm for 8 min. The supernatants were desalted using home‐made C18 stage tips (3 M Empore) filled with poros R3 (Applied Biosystems) resin. The stage tips were equilibrated with 80% acetonitrile (MeCN)/0.5% FA followed by 0.5% FA. Bound peptides were eluted with 30–80% MeCN/0.5% FA and lyophilized.

Dried peptide mixtures from each condition were resuspended in 200 mM Hepes, pH 8.3. TMT 1 lex/16plex reagent (Thermo Fisher Scientific), reconstituted according to manufacturer’s instruction, was added and incubated at room temperature for 1 h. The labeling reaction was then terminated by incubation with 5% hydroxylamine for 0.5 h. The labeled peptides were pooled into a single sample and desalted using the same stage tips method as above.

About 200 μg of the labeled peptides were separated on an off‐line, high pressure liquid chromatography (HPLC) using XBridge BEH130 C18, 5 µm, 2.1 × 150 mm (Waters) column with XBridge BEH C18 5 µm Van Guard Cartridge, connected to an Ultimate 3000 Nano/Capillary LC System (Dionex). Peptides were separated with a gradient of 1–90% B (A: 5% MeCN/10 mM ammonium bicarbonate, pH 8; B: MeCN/10 mM ammonium bicarbonate, pH 8, [9:1]) in 1 h at a flow rate of 250 µl/min. A total of 54 fractions were collected, combined into 18 fractions and lyophilised. Dried peptides were resuspended in 1% MeCN/0.5% FA and desalted using C18 stage tips and ready for mass spectrometry analysis.

The fractionated peptides were analyzed by LC‐MS/MS using a fully automated Ultimate 3000 RSLC nano System (Thermo Fisher Scientific) fitted with a 100 μm × 2 cm PepMap100 C18 nano trap column and a 75 μm × 25 cm, nanoEase M/Z HSS C18 T3 column (Waters). Peptides were separated using a binary gradient consisting of buffer A (2% MeCN, 0.1% FA) and buffer B (80% MeCN, 0.1% FA). Eluted peptides were introduced directly via a nanospray ion source into a Q Exactive Plus hybrid quardrupole‐Orbitrap mass spectrometer (Thermo Fisher Scientific). The mass spectrometer was operated in standard data‐dependent mode, performed survey full‐scan (MS, m/z = 380–1600) with a resolution of 70,000, followed by MS2 acquisitions of the 15 most intense ions with a resolution of 35,000 and NCE of 33% for TMT10plex (29% for TMTpro 16plex). MS target values of 3e6 and MS2 target values of 1e5 were used. Dynamic exclusion was enabled for 40 s.

The acquired raw files from LC‐MS/MS were processed using MaxQuant (Cox & Mann, 2008) with the integrated Andromeda search engine (v.1.6.6.0). MS/MS spectra were quantified with reporter ion MS2 from TMT 10plex and TMTpro 16plex experiments and searched against Human Reviewed and Mus musculus (downloaded in 2019), UniProt Fasta databases, respectively. Carbamidomethylation of cysteines was set as fixed modification, while methionine oxidation, N‐terminal acetylation, and STY phosphorylation were set as variable modifications. Protein quantification requirements were set at 1 unique and razor peptide. In the identification tap, second peptides and match between runs were not selected. Other parameters in MaxQuant were set to default values.

MaxQuant output file, proteinGroups.txt, and the Phospho (STY) sites.txt were then processed with Perseus software (v 1.6.6.0). After uploading the matrix, the data were filtered, to remove identifications from reverse database, identifications with modified peptide only, and common contaminants. The localization probability of phospho (STY).txt was also filtered to ≥ 0.75. Both sets of data with the reporter intensities of “0” were converted to NAN and exported as text file for further data analysis.

### Analysis of Ribo‐seq data

#### Initial data preparation

We obtained the RiboSeq FASTQ files described in (Misra *et al*, 2021) from the NCBI GEO database, deposited under the accession GSE156850. The data comprised 6 × 2 (150 bp) paired‐end FASTQ files, corresponding to 3 MEF cell samples treated with HF, and 3 untreated MEF cell controls. Preliminary quality control assessment was performed using FastQC (v0.11.5) (https://www.bioinformatics.babraham.ac.uk/projects/fastqc/).

The experimental protocol described in Misra *et al* necessitated that the RiboSeq data was pre‐processed prior to mapping because the sequence reads contained not only mouse‐derived DNA, but also non‐mouse DNA that needed to be removed from reads for effective mapping. To achieve this, a custom Python 3 script was written to filter FASTQ files by searching for the pre‐determined fixed sequences in each read, and thereby determining the 3‐prime end of each putative mouse sequence. The UMIs were not discarded completely by the script, but instead were moved into the FASTQ read headers, allowing for the subsequent identification of PCR duplicates.

While this pre‐processing aided mapping, identifying the fixed sequence also served as an additional quality control on the data, for only canonical reads should be expected to contain the fixed sequence. However, as base‐calling errors do occur during sequencing, the script allowed up to 3 mismatches between the putative fixed sequence in the read and expected target fixed sequence to prevent valid data being unnecessarily discarded.

The library was sequenced using a 150 bp paired‐end run, meaning that the reads were longer than the genetic construct of interest contained within them. Consequently, a canonical read‐pair should contain the same sequence, albeit reverse‐complemented, in both reads. For this reason, only forward reads (R1) were used in the subsequent analysis.

RiboSeq datasets frequently contain a high proportion of unwanted sequences, such as rRNA, which are best removed prior to mapping. To achieve this, we downloaded rRNA, tRNA, and mitochondrial mouse sequences from the NCBI *(* https://www.ncbi.nlm.nih.gov/nuccore *)* Nucleotide Database. Reads in the trimmed FASTQ files aligning to any of these rRNA, tRNA or mitochondrial sequences were removed using the filter functionality of FastQ Screen (version 0.14.1; Wingett & Andrews, 2018), running Bowtie 2 (version 2.3.4.1; Langmead & Salzberg, 2012).

#### Mapping

We aligned the trimmed FASTQ reads to both the GRCm38 (version 100) primary assembly and transcriptome reference genomes using the aligner STAR (version 2.5.4b; Dobin *et al*, 2013). STAR was run with the following parameters:

*STAR ‐‐runThreadN 8 ‐‐outFilterType Normal ‐‐outWigType wiggle ‐‐outWigStrand Stranded ‐‐outWigNorm RPM ‐‐alignEndsType EndToEnd ‐‐outFilterMismatchNmax 1 ‐‐outFilterMultimapNmax 1 ‐‐genomeDir [STAR Index Directory] ‐‐readFilesIn [FASTQ File] ‐‐outFileNamePrefix [File Prefix] ‐‐outSAMtype BAM SortedByCoordinate ‐‐quantMode TranscriptomeSAM GeneCounts ‐‐outSAMattributes All*

The resulting BAM files were sorted by genomic coordinate and indexed with Samtools (version 1.7; Li *et al*, 2009; *run* using htslib version 1.7‐2).

Following mapping, putative PCR duplicates (reads mapping to identical locations and sharing UMIs) were removed using UMI‐tools (version: 1.1.1; Smith *et al*, 2017). Before processing with UMI‐tools, the genome ID tags appended to every FASTQ read by FastQ Screen were removed using the *remove_tags.pl* Perl script, which is bundled with the FastQ Screen software.

#### RiboSeq analysis

We used the publicly available bioinformatics tool RiboMiner (version 0.2.3.1; Li *et al*, 2020) for the post‐mapping analysis. RiboMiner is a specialist tool designed for handling ribosome profiling data. Correct running of RiboMiner required the installation of other software dependencies, including RiboCode (version 1.2.11), which were all run on a Linux Mint 19 operating system.

#### Quality control

A standard ribosome profiling control is to verify the 3‐nt periodicity of the data for different read lengths. For this purpose, RiboMiner reports the distribution of ribosome protected fragments, aligned by their 5' end, relative to start and stop codons. Using this information, P‐site (and consequently A‐site) offsets may be determined for each read length. Read lengths exhibiting this expected 3‐nt periodicity were selected for subsequent analysis.

#### Ribosome density across transcripts

We used the *MetageneAnalysis* function of RiboMiner to calculate the read densities across transcripts (normalized by length). For genes that generate multiple transcripts, the longest transcript was selected by RiboMiner. The calculation was performed using the settings recommended in the RiboMiner documentation (https://github.com/xryanglab/RiboMiner).

In addition, we performed a similar analysis to assess the ribosome distribution bias for all genes using the RiboMiner *PolarityCalculation* function. Ribosome polarity can be considered conceptually similar to the “centre of mass” of the coverage profile. During the calculation, a value from −1 to 1 is assigned to each transcript. Negative values correspond to footprint enrichment at the 5′ end of a CDS, while conversely a positive score denotes 3′ enrichment. We ran the *PolarityCalculation* function using the settings recommended in the software documentation.

Importantly for comparative purposes, these two functions can take as an additional argument a list of gene IDs, to restrict analyses to a subset of genes—rather than using the whole genome.

#### Plotting ribosome density across specific individual genes

Reads mapped to the GRCm38 (version 100) primary assembly were imported into the genome browser and NGS analysis tool SeqMonk (version 1.48.0; https://www.bioinformatics.babraham.ac.uk/projects/seqmonk/). To assess the distribution of reads across a given gene, overlapping bins of 20 bp in length, with a step size of 1 bp, were generated and then quantitated by counting the number of reads overlapping each bin. The quantitated bins were reported in bedGraph format by SeqMonk, and those files were then imported into the Integrative Genomics Viewer (v2.10.0) software (Robinson *et al*, 2011) for visual examination and presentation purposes.

### Quantification and statistical analysis

Sample size for each experiment was determined based on previous studies.

The statistical analysis was performed using GraphPad Prism 8 using two‐tailed unpaired *t*‐test, one‐way or two‐way ANOVA or the nonlinear regression curve fit for inhibitory dose‐response (IC50). The data are presented as mean ± SD, unless stated otherwise. For proteomics data analysis, the reporter ion intensities were multiplied by global scaling factor in order to correct for sample loading differences between conditions. Differential protein abundance between groups was then assessed using the Bioconductor package edgeR (Robinson *et al*, 2010). The gene ontology (GO) analysis was performed using online tool DAVID (https://david.ncifcrf.gov/home.jsp; Huang *et al*, 2009a, 2009b).

## Author contributions

**Aleksandra P Pitera:** Conceptualization; Formal analysis; Validation; Investigation; Visualization; Writing—review and editing. **Maria Szaruga:** Formal analysis; Validation; Investigation; Visualization; Writing—review and editing. **Sew‐Yeu Peak‐Chew:** Investigation; Writing—review and editing. **Steven W Wingett:** Software; Formal analysis; Writing—review and editing. **Anne Bertolotti:** Conceptualization; Supervision; Funding acquisition; Writing—original draft; Project administration; Writing—review and editing.

In addition to the CRediT author contributions listed above, the contributions in detail are:

AB and APP designed the study. APP conducted all experiments. MS designed and conducted experiments presented in Fig 7. S‐Y P‐C performed the quantitative proteomic experiments. SWW performed the computational analyses of ribosome profiling. APP, MS, and SWW prepared the figures. AB wrote the paper with support from APP and S‐YP‐C and SWW for the proteomic and computational methods, respectively.

## Disclosure and competing interests statement

AB is an editorial advisory board/EMBO Member. This has no bearing on the editorial consideration of this article for publication.

## Supporting information



Expanded View Figures PDF

Dataset EV1

Dataset EV2

## Data Availability

The mass spectrometry proteomics data have been deposited to the ProteomeXchange Consortium via the PRIDE (Perez‐Riverol *et al*, 2019) partner repository with the dataset identifier PXD028744 (http://www.ebi.ac.uk/pride/archive/projects/PXD028744).
