# Supplementary material for: Cellular responses to halofuginone reveal a vulnerability of the GCN2 branch of the integrated stress response
Source: EMBO J. 2022 Apr 25;41(11):e109985. doi: 10.15252/embj.2021109985 (PMC9156968; doi:10.15252/embj.2021109985)
Supplement: Supplementary file 1 — Expanded View Figures PDF [file EMBJ-41-e109985-s001.pdf]

Expanded View Figures

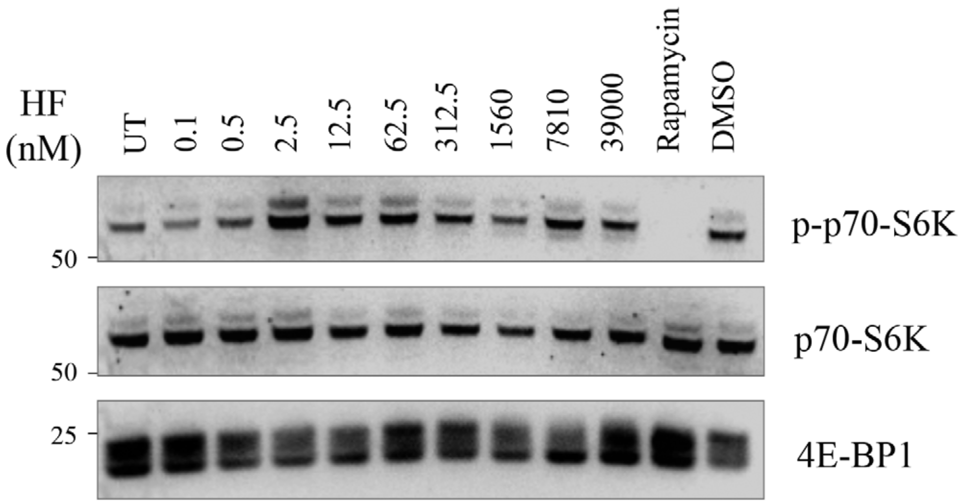

Figure EV1. mTORC1 activation upon HF treatment.

Representative immunoblots of indicated proteins in lysates from HeLa cells treated for 5 h with indicated concentrations of HF or 200 nM Rapamycin for 3 h.

© 2022 MRC Laboratory of Molecular Biology

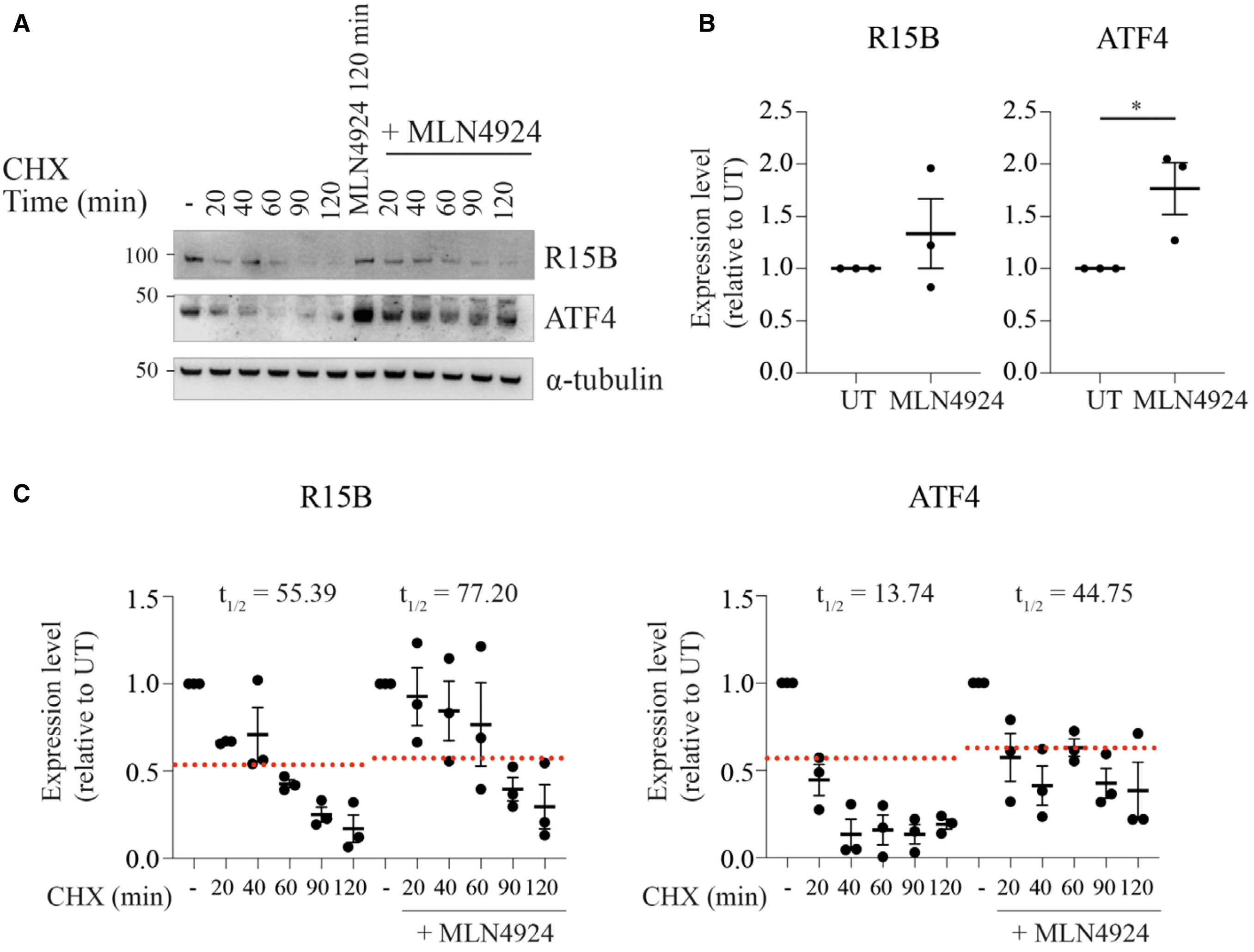

Figure EV2. The Nedd8-activating enzyme inhibitor MLN4924 stabilizes ATF4 and R15B.

A Representative immunoblots of the indicated proteins in lysates from HeLa cells treated with cycloheximide (20 μg/ml) with or without the inhibitor of the NEDD8-activating enzyme MLN4924 (1 μM) for indicated times.

B Quantification of R15B and ATF4 in cells treated with MLN4924 (1 μM) for 120 min. Data are mean ± SEM (n = 3 biological replicates). *P = 0.0369, as determined by unpaired t-test.

C Quantification of experiments such as the ones shown in (A). $t_{1/2}$ values were calculated with results from 3 independent experiments. Data are mean ± SEM (n = 3 biological replicates).

 © 2022 MRC Laboratory of Molecular Biology

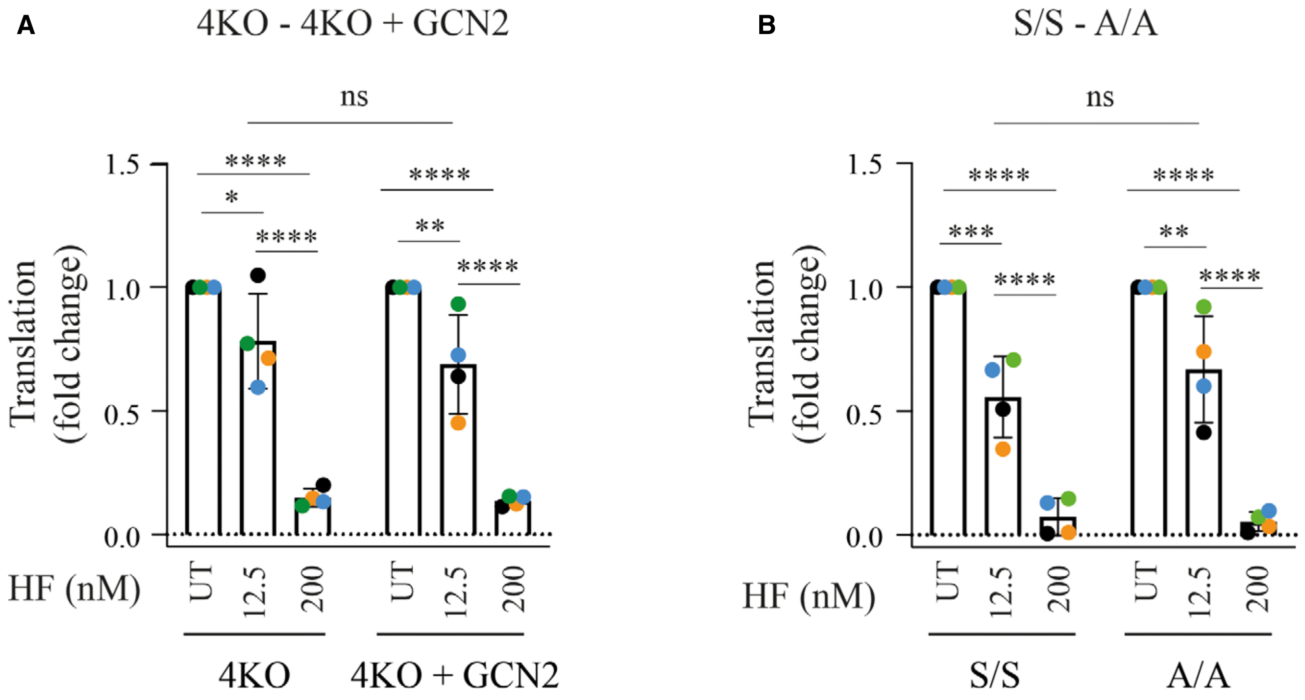

Figure EV3. Translational attenuation following HF is independent of GCN2 or eIF2α phosphorylation.

A, B Quantification of ^{35}S-methionine labeling in experiments such as the ones shown in Fig 6C and D, respectively. Data are mean ± SD ($n \geq 3$ biological replicates). *$P \leq 0.0376$, **$P \leq 0.0031$, ***$P \leq 0.0001$, ****$P < 0.0001$, ns: not significant, as determined by two-way ANOVA.

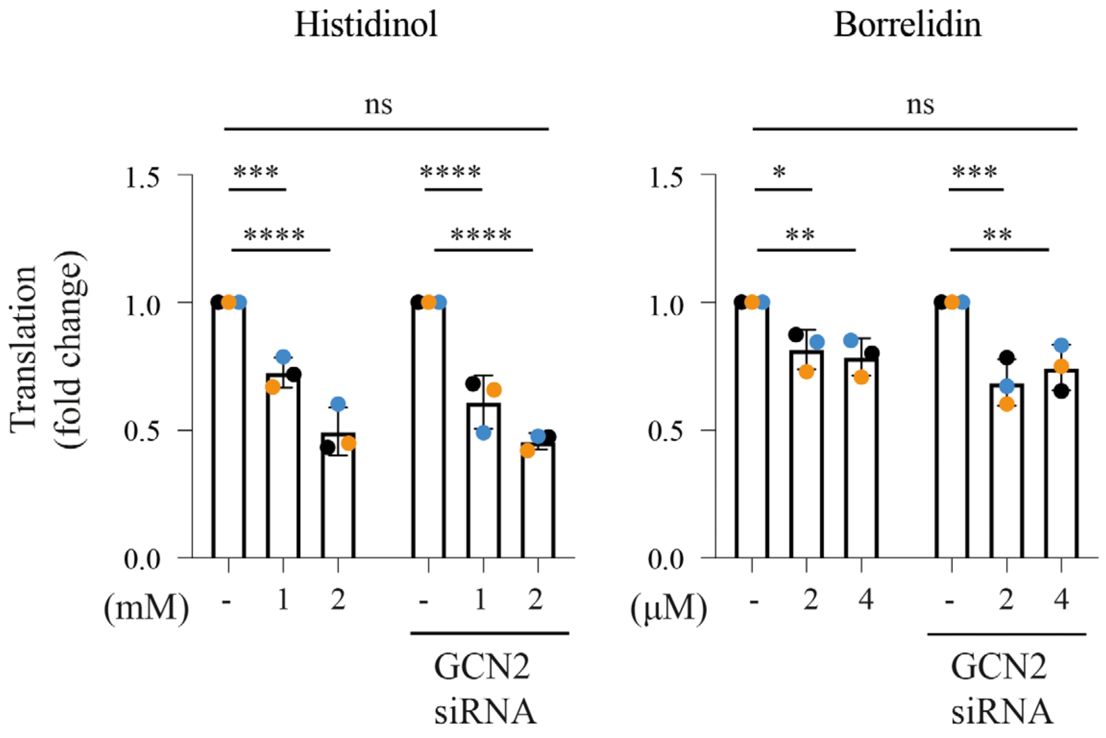

Figure EV4.

Figure EV4. Translational attenuation following histidinol or borrelidin is independent of GCN2.

Quantification of ^{35}S-methionine labeling in experiments such as the ones shown in Fig 6I. Data are mean ± SD (n = 3 biological replicates). *$P \leq 0.0116$, **$P \leq 0.0044$, ***$P \leq 0.0004$, ****$P < 0.0001$, ns: not significant, as determined by two-way ANOVA.

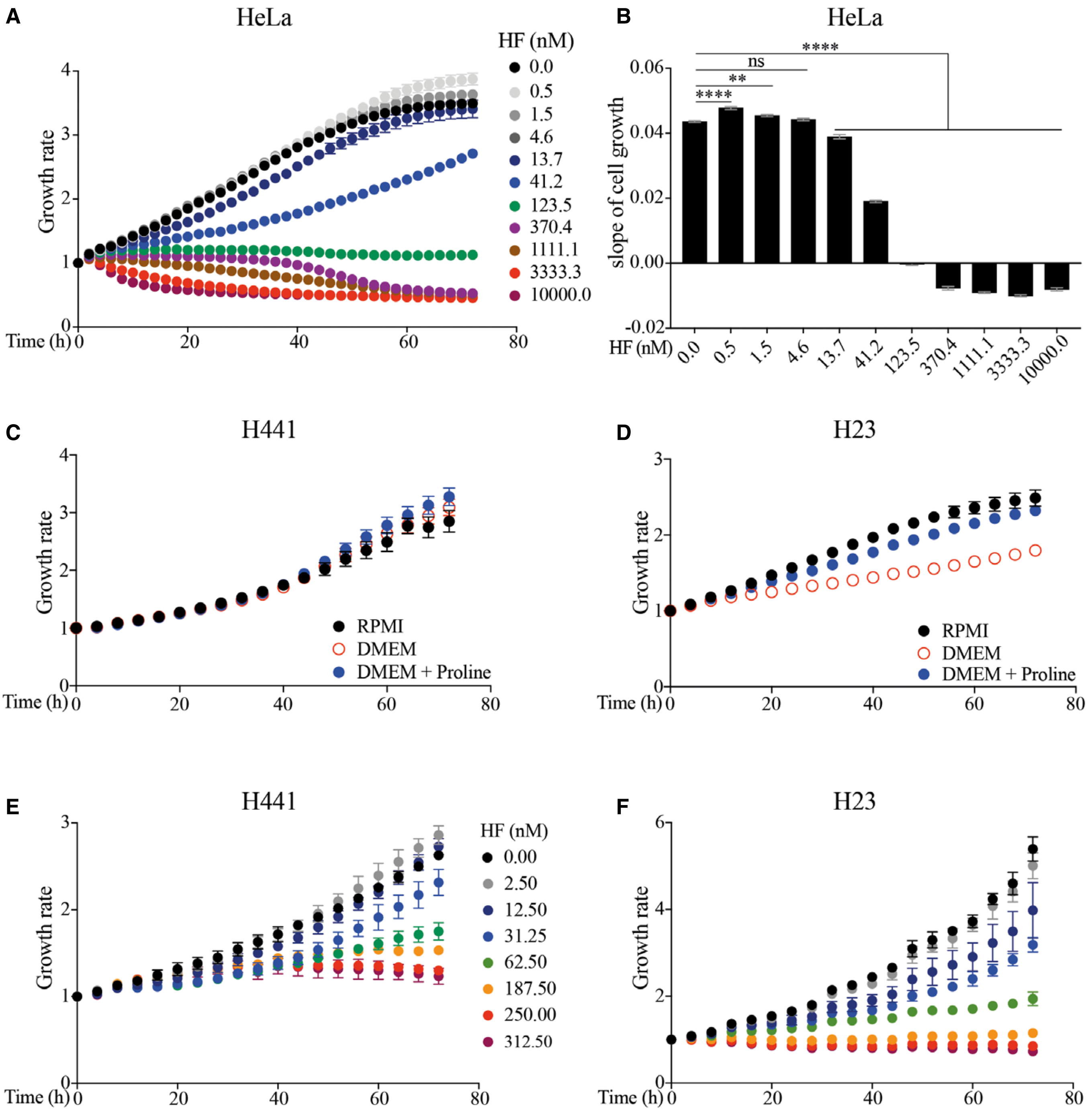

Figure EV5.

© 2022 MRC Laboratory of Molecular Biology

Figure EV5. Differential effect of HF on growth rates of HeLa, H441 and H23 cells.

A, B Growth rates (A) and the corresponding slopes of cell growth (B) of HeLa cells treated with indicated concentrations of HF and monitored for 72 h at 37°C in an Incucyte S3 system. Slope of cell growth was determined by simple linear regression curve fit of exponential cellular growth rate (0–56 h) in distinct conditions. Data are mean ($n \geq 4$ biological replicates) ± SEM. $^{**}P = 0.0073$, $^{****}P < 0.0001$, as determined by one-way ANOVA with Dunnett's multiple comparison test. ns: not significant.

C, D Growth rates of H441 (C) and H23 (D) lung cancer cell lines grown for 72 h at 37°C in an Incucyte S3 system in RPMI (containing 20 mg/l Proline) media versus DMEM (Proline-free) or DMEM supplemented with 20 mg/l Proline. Data are mean ($n = 4$ biological replicates) ± SEM.

E, F Growth rates of H441 (E) and H23 (F) cells exposed to indicated concentrations of HF for 72 h at 37°C in an Incucyte S3 system. Data are mean ($n = 3$ biological replicates) ± SD.

© 2022 MRC Laboratory of Molecular Biology
