# Supplementary material for: Cellular responses to halofuginone reveal a vulnerability of the GCN2 branch of the integrated stress response
Source: EMBO J. 2022 Apr 25;41(11):e109985. doi: 10.15252/embj.2021109985 (PMC9156968; doi:10.15252/embj.2021109985)
Supplement: Supplementary file 4 [file EMBJ-41-e109985-s004.pdf]

THE EMBO JOURNAL

Cellular responses to halofuginone reveal a vulnerability of the GCN2 branch of the integrated stress response

Aleksandra Pitera, Maria Szaruga, Sew-Yeu Peak-Chew, Steven Wingett, and Anne Bertolotti
DOI: 10.15252/embj.2021109985

Corresponding author(s): Anne Bertolotti (aberto@mrc-lmb.cam.ac.uk)

Review Timeline:

Submission Date:	19th Oct 21
Editorial Decision:	14th Dec 21
Revision Received:	11th Feb 22
Editorial Decision:	10th Mar 22
Revision Received:	16th Mar 22
Accepted:	26th Mar 22

Editor: Stefanie Boehm

Transaction Report:

(Note: With the exception of the correction of typographical or spelling errors that could be a source of ambiguity, letters and reports are not edited. Depending on transfer agreements, referee reports obtained elsewhere may or may not be included in this compilation. Referee reports are anonymous unless the Referee chooses to sign their reports.)

Thank you again for submitting your manuscript EMBOJ-2021-109985 for consideration by The EMBO Journal. Please also excuse the delay in communicating this decision to you, which was due to a repeatedly delayed referee report, as previously mentioned. Despite having been in contact with the referee, we still have not received this report, and in the interest of time we have decided to nonetheless proceed and make a preliminary decision based on the two reports at hand (copied below for your information).

As you will see, these two reviewers raise several overlapping points, but do not agree in their overall assessment of the study, with referee #1 being negative and referee #3 more positive. Therefore, I again contacted referee #3 and asked her/him to comment specifically on the other referee's concerns. Given her/his response, emphasizing the overall interest of the findings and indicating that several issues may well be addressable, we have decided to give you the opportunity to respond to the raised issues in a revised version of the manuscript. For such a revision to be successful, it will however be important to fully resolve all of referee #3's concerns, in particular those that overlap with referee #1. Please also respond to all other comments of referee #1 and address these experimentally or by textual revision.

Given this specific situation with two contradicting reviewer opinions on several aspects of the study, please also be aware that we may need to involve an additional arbitrating expert to help assess the revised version. We can only consider the manuscript further if the crucial issues are resolved, which we realize will likely involve a substantial experimental revision. Therefore, I would encourage you to carefully consider the referee points and to contact me with a preliminary point-by-point response to a discuss a revision plan as soon as possible. The aim of such a discussion would be to identify potential issues as early as possible and to ensure that the crucial points are being addressed.

Referee #1:

This paper attempts to demonstrate a supposed vulnerability of the ISR to inhibition of prolyl tRNA charging by the drug HF. The experiments show that intermediate concentrations of HF (12-62 nM) that evoke moderate reductions in bulk protein synthesis of 20-50% (Fig. 6) confer robust induction of the ISR, increasing Atf4 protein expression more than that of ATF4 mRNA levels, consistent with translational activation of ATF4 mRNA (Fig. 1), in a manner dependent on eIF2α phosphorylation on Ser51 (Fig. 3A). As expected, the increased eIF2α phosphorylation evoked by HF is dependent on the kinase Gcn2, as shown previously by others; and they show convincingly that none of the other 3 ISR kinases will suffice. All of this is consistent with expectations as Gcn2 is known to be activated by amino acid limitation or defects in tRNA charging. The authors focus on the fact that at higher concentrations of HF the Atf4 protein is not induced, despite an abundance of its mRNA and high levels of phosphorylated eIF2. They make a similar finding for the translationally induced R15A subunit of phosphatase PP1 that dephosphorylates eIF2. Accumulation of the constitutively expressed R15B subunit of PP1 is also reduced at high HF concentrations. They carry out some rather problematic experiments aimed at showing that Atf4 and R15B are not being degraded more rapidly at high HF concentrations, and thus conclude that these proteins decline owing entirely to their reduced synthesis. They interrogate published ribosome profiling data on HF treatment and find evidence for reduced elongation rates in the metagene analysis of ribosome occupancies across coding sequences, consistent with the previous evidence for ribosome stalling at proline codons in HF treated cells in the manner expected for reduced formation of charged Prolyl tRNA. Measurements of labeled methionine incorporation show that bulk protein synthesis is indeed inhibited at high concentrations of HF; and this occurs similarly in the presence or absence of Gcn2 or phosphorylatable eIF2α, which the authors find surprising since activated Gcn2 should be contributing to a reduction in translation at the initiation step. The effects of high HF on bulk translation and accumulation of Atf4 and R15B can be reversed by proline, as expected if HF impedes charging of Prolyl tRNA. They conclude that the inhibition of translation elongation at high HF concentrations prevents the induction of Atf4 synthesis despite high-level eIF2α phosphorylation. In addition, the finding that the inhibition of bulk translation by HF appears seems to occur equally in the presence or absence of Gcn2 and eIF2 phosphorylation prompts them to infer a "vulnerability" of the ISR to HF, because the ISR cannot prevent the inhibition of elongation in response to a strong reduction in prolyl tRNA charging at elevated HF levels.

They finish by showing that proliferation of certain tumor cells is dependent on exogenous proline in the medium and that these particular tumor cells are hypersensitive to HF, suggesting a possible therapeutic application for HF. They characterize this last finding as a consequence of the putative vulnerability of the ISR to HF. However, this strikes me as an overinterpretation of the simple fact that these particular tumor cells have a heightened requirement for proline, so of course they would be hypersensitive to HF whose inhibitory effects on prolyl tRNA charging scale with proline availability in the cells.

The fact that Atf4 cannot be induced at high HF concentrations despite high-level eIF2 phophorylation is not surprising because Atf4, like the majority of proteins, requires efficient translation of proline codons. At intermediate concentrations of HF, Atf4 synthesis can be induced because the inhibition of elongation is modest, judging by the small reductions in bulk protein synthesis observed at these concentrations. I believe that the authors concur with this general interpretation. As noted above, what captures their attention are their findings that seem to indicate that inhibition of bulk translation at the elongation stage by HF occurs to the same extent in the presence or absence of the Gcn2/eIF2 pathway, suggesting to them that the Gcn2/eIF2

pathway fails to attenuate the initiation rate in the manner required to prevent translation elongation defects. There are multiple problems here. (i) It is unrealistic, and amounts to "putting up a straw man", to suggest that attenuating initiation would be able to prevent a strong inhibition of translation elongation at the high concentrations of HF that block prolyl tRNA charging and dramatically slow the rate of decoding proline codons. (ii) They have not actually shown that Gcn2 does not inhibit initiation at elevated HF concentrations. They have measured only the overall protein synthesis rates, not the separate rates of initiation and elongation that in combination determine the overall rate of translation. It is possible that when Gcn2 is present it is reducing the initiation rate owing to high-level eIF2 phosphorylation and that this slows the consumption of proline to diminish the effect of HF in reducing prolyl tRNA charging and the attendant reduction in elongation at proline codons. When Gcn2 is absent, there is no inhibition of initiation and so HF can more effectively impede prolyl tRNA charging and elongation. As a result, the net reduction in protein synthesis can be similar in the presence or absence of Gcn2, but owing to differential effects on initiation versus elongation. Unless they can separately assess initiation and elongation rates +/- Gcn2 in the presence of high-level HF, it would seem that they cannot discard this alternative explanations. (iii) Much of the evidence presented that the reduction in translation conferred by high level HF is the same in the presence or absence of Gcn2 is not convincing. The results in Fig. 6C-D suffer from the fact that at the high HF concentrations employed there, the inhibition of translation is nearly complete in the absence of Gcn2, making it difficult to determine whether it is being reduced to a greater extent when Gcn2 is present. At these high HF concentrations, elongation through proline codons may be so rate-limiting that an additional reduction in the initiation rate will have little impact on the overall rate of translation. The experiment in Fig. 6F is better, as the HF of 62.5 nM is reducing translation in the absence of Gcn2 by only 45%. Although it seems to be reduced to nearly the same extent in the presence of Gcn2, the loading of the gel is not even, and the reduction in translation with Gcn2 knocked-down may be underestimated. (iv) Even if we assume that the authors are correct, why would the reduced charging of prolyl-tRNA conferred by HF interfere with the inhibition of initiation by eIF2 phosphorylation? And why should there by anything special about HF that would not be seen at high concentrations of other inhibitors of aminoacyl tRNA synthetases, or severe amino acid limitation imposed by an inhibitor of amino acid biosynthesis in medium lacking the amino acid? (v) The authors like to contrast the effect of HF with activation of PERK by tunicamycin (Tm), but this is comparing apples to oranges as TM only inhibits initiation by activating PERK while HF has the dual effect of inhibiting initiation via Gcn2 at low concentrations and also inhibiting elongation at high concentrations; and does not seem surprising that the latter effect would become rate-limiting, and therefore insensitive to reduced initiation at high concentrations of HF.

-In addition to these fundamental issues, the experiment in Fig. 4D-E is quite flawed. The error bars are huge for R15B making it unlikely that an increase in t1/2 of only 30% can be statistically significant. For Atf4, degradation still occurs rapidly in the presence of MLN4924. In fact, the initial rate appears to be faster with MLN present, which would be evident if the data was plotted as the fraction of protein at time zero that remains at each time point after CHX addition. Furthermore, MLN is inexplicably inducing ATF4 expression prior to CHX addition. These complications undermine their attempts to show that degradation of these proteins is substantially altered by MLN. This in turn undermines the conclusions made from the experiments in Fig. 4F. Even if we accepted their interpretation of Fig. 4F, that the reduction in R15B and Atf4 levels still occur when TRCP-dependent degradation is inhibited, it could still be the case that enhanced degradation is occurring by a separate, TRCP-independent pathway that operates in HF-treated cells.

Other points:

-The sentence on p. 8 "R15A induction was not detected in these global proteomic analyses (Figure 3 and Table EV1), although it was manifest on immunoblot analyses (Figure 1A and 1D), most probably because of its low abundance (http://mapofthecell.biochem.mpg.de/) (Itzhak et al., 2016)" ignores the fact that the concentration of HF of 12.5 was too low to induce R15A in Fig. 1A.

-p. 11 and Fig. 5A: they should present the polarity scores for the induced and repressed genes to bolster their conclusions that only the HF-repressed genes show a shift in ribosome density towards the 5' end.

-p. 11: The sentence "The translationally repressed mRNAs displayed an increased ribosome density in their 5' UTR (Figure 5D)" is confusing because the legend seems to indicate that the metagene plots show ribosome occupancies beginning at the start codons of the main coding sequences. As such, they do not report on 5'UTR translation. This is very confusing.

Referee #3:

This is an interesting and well executed study. The authors find that attenuation of ATF4 induction by high doses of halofuginone is caused by translation elongation defects rather than by eIF2alpha kinase regulation. The results are consistent with work cited by the authors showing that ribosome stalling due to low tRNA charging is sufficient to reduce global and gene-specific protein synthesis, and that loss of the GCN2 response to ribosome stalling/uncharged tRNA exacerbates this phenomenon. The authors should consider the following points prior to publication.

1. Figure 4D-F is not particularly convincing. Though MLN4924 clearly affects the baseline level of R15B and Atf4, the proteins are degraded at the same rate or even faster in the presence of MLN4924. Specifically, the slope of the line in 4E is similar or more steep, indicating faster degradation, in the presence of MLN4924. This misinterpretation may be caused by using the 50% level of the protein in the UT condition only to calculate the half life +/- MLN4924, which is misleading because the initial level is so much higher in the presence of the drug. Instead, the 50% level of the protein separately +/- drug should be used to calculate its half life. The real half life of ATF4 +MLN4924 looks to be ~18 minutes, and the real half life of R15B +MLN4924 looks to be ~60 minutes, suggesting that they are degraded similarly in the presence and absence of MLN4924. The data suggest that MLN4924 has little effect on the degradation rate of these proteins, but perhaps increases their expression at baseline. An

potentially more convincing way to show that degradation does not explain reduced Atf4/R15B levels at high HF doses, which is important, would be to use a more general proteasome inhibitor. I think it would only be necessary panel F in the presence of a proteasome inhibitor.

2. The growth rate data in Figure 7 is difficult to interpret as shown. In any condition where the cells reach 100% confluency after 72 hours, any real growth rate differences before that point are masked because it is unclear when the cells became saturated. For example, one condition could have reached confluency at 48 hours and another at 72 hours, presenting a significant growth rate alteration, and the data would look the same at 72 hours by this measurement. Based on the methods section it seems that the authors have time course data from the IncuCyte that should address this issue. Rather than ,or in addition to, showing % confluency at the end, they should show the full growth curve, and/or calculate the slope from the portion of the curve where cells are in exponential growth.

Minor points/suggestions:

1. Can the authors comment on why Atf4/R15A mRNA levels are independent of Atf4 levels?

2. It may be interesting to correlate the log2 fold changes in figure 3 with the absolute proline content of each protein to further support a ribosome stalling based mechanism for translational repression.

3. The authors might point out in Figure 5D that increased ribosome density in the coding region of Atf4 could also be caused by slow elongation/ribosome stalling - so it is difficult to interpret this signal, and it does not necessarily indicate increased Atf4 production (in fact it is unclear to me what to expect because 100 nM is around the inflection point for their observed attenuation of Atf4 induction by HF between 62.5 and 200-300 nM). It would be interesting to also show this plot for R15B.

4. The wording of the results description of Figure 4C is confusing - I think the authors mean not statistically significantly different between 4KO and 4KO+GCN2, as they indicate for the description of 4D.

5. It may be interesting to speculate that eIF2alpha phosphorylation continues to increase at high HF doses because R15A/B expression are attenuated. However, this could also result from increased activation of GCN2 at high HF doses due to lower tRNA charging and more ribosome collisions. This could be discussed.

Referee #1:

We thank the reviewer for his detailed comments on the manuscript. We have edited the text where required and added new datasets that strengthen the study.

This paper attempts to demonstrate a supposed vulnerability of the ISR to inhibition of prolyl tRNA charging by the drug HF.
Our response: This is incorrect. We did not suppose a vulnerability of the ISR. We were as surprised as this reviewer by the findings. The study aimed at understanding why HF induced an atypical ISR (loss of ATF4 induction at concentrations of HF above 312.5 nM). We searched for the underlying mechanism(s) in unbiased ways, conducting time courses, dose responses, proteomic analyses and lastly, examined translation. We were sceptical at first to see no measurable impact of GCN2 on translation attenuation at any concentration of HF. This prompted us to challenge our observations in various orthogonal experimental systems: GCN2 knock-out cells, eIF2α$^{A/A}$ cells and cells with GCN2 knockdown. The findings were similar in all systems tested. Thus, our conclusion is robust and unanticipated.

The experiments show that intermediate concentrations of HF (12-62 nM) that evoke moderate reductions in bulk protein synthesis of 20-50% (Fig. 6) confer robust induction of the ISR, increasing Atf4 protein expression more than that of ATF4 mRNA levels, consistent with translational activation of ATF4 mRNA (Fig. 1), in a manner dependent on eIF2α phosphorylation on Ser51 (Fig. 3A). As expected, the increased eIF2α phosphorylation evoked by HF is dependent on the kinase Gcn2, as shown previously by others; and they show convincingly that none of the other 3 ISR kinases will suffice. All of this is consistent with expectations as Gcn2 is known to be activated by amino acid limitation or defects in tRNA charging. The authors focus on the fact that at higher concentrations of HF the Atf4 protein is not induced, despite an abundance of its mRNA and high levels of phosphorylated eIF2. They make a similar finding for the translationally induced R15A subunit of phosphatase PP1 that dephosphorylates eIF2. Accumulation of the constitutively expressed R15B subunit of PP1 is also reduced at high HF concentrations. They carry out some rather problematic experiments aimed at showing that Atf4 and R15B are not being degraded more rapidly at high HF concentrations, and thus conclude that these proteins decline owing entirely to their reduced synthesis.

Our response: Part of the experiments criticized here, half-life of R15B and ATF4 in the presence of the Nedd8 inhibitor MLN4924, consists of a repetition of published data. These experiments have now been moved to the EV section of the manuscript. We have added new experiments that confirm that degradation is not responsible for the loss of ATF4 and R15B upon HF. We have now carried out experiments with two different inhibitors of degradation (MLN4924 and MG-132, Figure 4D and 4E, respectively). Thus, the results are robust and confirmed our conclusions (more below).

They interrogate published ribosome profiling data on HF treatment and find evidence for reduced elongation rates in the metagene analysis of ribosome occupancies across coding sequences, consistent with the previous evidence for ribosome stalling at proline codons in HF treated cells in the manner expected for reduced formation of charged Prolyl tRNA. Measurements of labeled methionine incorporation show that bulk protein synthesis is indeed inhibited at high concentrations of HF; and this occurs similarly in the presence or absence of Gcn2 or phosphorylatable eIF2α, which the authors find surprising since activated Gcn2 should be contributing to a reduction in translation at the initiation step. The effects of high HF on bulk translation and accumulation of Atf4 and R15B can be reversed by proline, as expected if HF impedes charging of Prolyl tRNA. They conclude that the inhibition of translation elongation at high HF concentrations prevents the induction of Atf4 synthesis despite high-level eIF2α phosphorylation. In addition, the finding that the inhibition of bulk translation by HF appears seems to occur equally in the presence or absence of Gcn2 and eIF2 phosphorylation prompts them to infer a "vulnerability" of the ISR to HF, because the ISR cannot prevent the inhibition of elongation in response to a strong reduction in prolyl tRNA charging at elevated HF levels. They

finish by showing that proliferation of certain tumor cells is dependent on exogenous proline in the medium and that these particular tumor cells are hypersensitive to HF, suggesting a possible therapeutic application for HF. They characterize this last finding as a consequence of the putative vulnerability of the ISR to HF. However, this strikes me as an overinterpretation of the simple fact that these particular tumor cells have a heightened requirement for proline, so of course they would be hypersensitive to HF whose inhibitory effects on prolyl tRNA charging scale with proline availability in the cells.
Our response: We don't think that we have overinterpreted the data. It is important to bring some context here. It has been known since 2012 (PMID: 22327401) that HF inhibits proline t-RNA synthetase and yet no one has tested/reported the sensitivity of proline-addicted cells to HF during this past 10 years. Thus, the knowledge that some cancer cells depend on proline and HF inhibits EPRS was not sufficient to prompt others to test the sensitivity of HF on proline-dependent cells.

The fact that Atf4 cannot be induced at high HF concentrations despite high-level eIF2 phophorylation is not surprising because Atf4, like the majority of proteins, requires efficient translation of proline codons. At intermediate concentrations of HF, Atf4 synthesis can be induced because the inhibition of elongation is modest, judging by the small reductions in bulk protein synthesis observed at these concentrations. I believe that the authors concur with this general interpretation. As noted above, what captures their attention are their findings that seem to indicate that inhibition of bulk translation at the elongation stage by HF occurs to the same extent in the presence or absence of the Gcn2/eIF2 pathway, suggesting to them that the Gcn2/eIF2 pathway fails to attenuate the initiation rate in the manner required to prevent translation elongation defects.

There are multiple problems here. (i) It is unrealistic, and amounts to "putting up a straw man", to suggest that attenuating initiation would be able to prevent a strong inhibition of translation elongation at the high concentrations of HF that block prolyl tRNA charging and dramatically slow the rate of decoding proline codons.
Our response: The experiments presented here were **not only conducted at high concentrations** where translation inhibition is strong **but over a broad range of concentrations, including low concentrations where translation inhibition is ~20%.** At 12.5 nM of HF, translation is reduced by ~20% in ISR-competent cells (Figure 6A, 6C, 6D). At 62.5 nM HF translation is reduced by ~ 40-50% (Figure 6A, 6F). As a benchmark, we used Tunicamycin and showed that it reduced translation by 30-40% after 2.5h (Figure 6E). We found that this effect is abolished in the eIF2$\alpha^{A/A}$ cells (Figure 6E). This establishes that the experimental system enables detection of small translation attenuation (20-40%) and the abrogation of this effect in an eIF2$\alpha^{A/A}$ cells. Thus, the lack of measurable effect of absence of the Gcn2/eIF2α pathway is not due to using high concentrations of HF. We have shown this with very low decrease of translation and low concentrations of HF.

(ii) They have not actually shown that Gcn2 does not inhibit initiation at elevated HF concentrations. They have measured only the overall protein synthesis rates, not the separate rates of initiation and elongation that in combination determine the overall rate of translation. It is possible that when Gcn2 is present it is reducing the initiation rate owing to high-level eIF2 phosphorylation and that this slows the consumption of proline to diminish the effect of HF in reducing prolyl tRNA charging and the attendant reduction in elongation at proline codons. When Gcn2 is absent, there is no inhibition of initiation and so HF can more effectively impede prolyl tRNA charging and elongation. As a result, the net reduction in protein synthesis can be similar in the presence or absence of Gcn2, but owing to differential effects on initiation versus elongation. Unless they can separately assess initiation and elongation rates +/- Gcn2 in the presence of high-level HF, it would seem that they cannot discard this alternative explanations.
Our response: The idea that attenuation of translation initiation occurs via GCN2 in wild-type cells to a degree that is exactly matched by translation elongation defects in absence of a functional ISR to result in the same net reduction of protein synthesis, over a range of concentrations, in diverse experimental systems, is improbable.

However, we have reviewed and edited the manuscript to ensure that we don't claim that GCN2 does not decrease translation initiation as indeed we don't measure translation initiation.

We see no measurable impact of the GCN2-eIF2α signalling pathway on translation upon HF. This is a surprising finding, fully supported by data in multiple independent systems. Importantly, this occurs over a broad range of concentrations, not just at high concentrations of HF.

(iii) Much of the evidence presented that the reduction in translation conferred by high level HF is the same in the presence or absence of Gcn2 is not convincing. The results in Fig. 6C-D suffer from the fact that at the high HF concentrations employed there, the inhibition of translation is nearly complete in the absence of Gcn2, making it difficult to determine whether it is being reduced to a greater extent when Gcn2 is present. At these high HF concentrations, elongation through proline codons may be so rate-limiting that an additional reduction in the initiation rate will have little impact on the overall rate of translation.
Our response: We disagree. Here again the reviewer focuses on experiments done at high concentrations whilst the paper focuses on findings made using low concentrations (12.5 (and 62.5) nM of HF in Figure 6A, 6C, 6D, 6F) leading to low rates of translation decrease (20-40%). We were as sceptical as the reviewer when we first saw that translation attenuation was not measurably different in the absence of GCN2 that we challenged these findings. In different experimental systems tested here (GCN2 knock-out cells, eIF2$\alpha^{A/A}$ cells and cells with GCN2 knockdown) and over a range of concentrations (12.5-312.5 nM for HeLa and 12.5- 200 nM for MEF cells), translation attenuation (20-40%) is not different with or without GCN2-eIF2α. The data is solid, and unexpected.

We do understand reviewer's point regarding high HF concentrations completely blocking translation. This is why we investigated lower concentrations of HF that result in ISR induction (Figure 1, 2, 3) and without causing a general loss of proteins that is seen at high HF concentration due to translation block (Figure 3).

In agreement with the reviewer's point, we show indeed that, at high HF concentration inhibition of translation is nearly complete in the absence as well as in the presence of GCN2 (Figure 6A, 6C, 6D and 6F). However, we conducted a large amount of work at low concentrations, which makes it possible to determine the effect (or its absence) of GCN2. Thus, the reviewer's issue is negated by the data summarized here. In 4KO cells, upon 12.5 nM HF, translation was at 77%, whereas in 4KO + GCN2, translation was at 93% (Figure 6C). Repetitions of this experiment did not reveal any significant difference between the cell lines (Figure EV3). Similarly to 4KO cells, in A/A cells treated with 12.5nM HF translation was attenuated by 40%, while in S/S cells this attenuation was of 33% (Figure 6D). Again, statistical analysis of multiple experiments did not reveal any difference meaning there is no measurable contribution of phosphorylated eIF2α to the HF-induced translational attenuation (figure EV3). It was such a surprise to us that we decided to assess our ability to detect ISR-dependent translational attenuation and its ISR-dependency (Figure 6E). Finally, we performed similar experiments in yet another system, GCN2 knockdown in HeLa cells, upon treatment of with low (12.5 nM), intermediate (62.5 nM) and high (312.5 nM) HF concentrations and confirmed previous findings (Figure 6F).

The experiment in Fig. 6F is better, as the HF of 62.5 nM is reducing translation in the absence of Gcn2 by only 45%. Although it seems to be reduced to nearly the same extent in the presence of Gcn2, the loading of the gel is not even, and the reduction in translation with Gcn2 knocked-down may be underestimated. (iv) Even if we assume that the authors are correct, why would the reduced charging of prolyl-tRNA conferred by HF interfere with the inhibition of initiation by eIF2 phosphorylation? And why should there by anything special about HF that would not be seen at high concentrations of other inhibitors of aminoacyl tRNA synthetases, or severe amino acid limitation imposed by an inhibitor of amino acid biosynthesis in medium lacking the amino acid?
Our response: In our discussion, we mention that "*The findings reported here using HF … may have relevance to the response to amino acid limitation and tRNA synthetase deficiencies in mammals.*"

Supporting this possibility, "*arginine deprivation has been reported to decrease translation partly through GCN2 and partly through ribosome pausing (Darnell et al., 2018)*."
Thus, what we describe here may have broad relevance. To support this point, we include in the revised version datasets showing that the inhibitor of histidine tRNA-charging histidinol as well as the threonine-tRNA synthetase inhibitor borrelidin also caused translation attenuation to a similar magnitude in wild-type cells and in cells where GCN2 was knocked-down (New Figure 6I and New EV4).

(v) The authors like to contrast the effect of HF with activation of PERK by tunicamycin (Tm), but this is comparing apples to oranges as TM only inhibits initiation by activating PERK while HF has the dual effect of inhibiting initiation via Gcn2 at low concentrations and also inhibiting elongation at high concentrations; and does not seem surprising that the latter effect would become rate-limiting, and therefore insensitive to reduced initiation at high concentrations of HF.
Our response: Firstly, we would like to remind the reviewer that the experiments were not only conducted at high concentrations of HF but also at low concentrations (12.5, 62.5 nM etc) throughout the study with GCN2 having no measurable effect on translation at any concentrations.
Regarding the PERK and GCN2 comparison to apples and oranges, this is precisely the point of the manuscript. In light of the findings presented here, GCN2 and PERK signalling appear like apples and oranges, whilst one expected that the two kinases had similar output. This is in fact why it has been named "integrated stress response". We show that the responses are different. This is an important finding.

-In addition to these fundamental issues, the experiment in Fig. 4D-E is quite flawed. The error bars are huge for R15B making it unlikely that an increase in t1/2 of only 30% can be statistically significant. For Atf4, degradation still occurs rapidly in the presence of MLN4924. In fact, the initial rate appears to be faster with MLN present, which would be evident if the data was plotted as the fraction of protein at time zero that remains at each time point after CHX addition. Furthermore, MLN is inexplicably inducing ATF4 expression prior to CHX addition. These complications undermine their attempts to show that degradation of these proteins is substantially altered by MLN. This in turn undermines the conclusions made from the experiments in Fig. 4F. Even if we accepted their interpretation of Fig. 4F, that the reduction in R15B and Atf4 levels still occur when TRCP-dependent degradation is inhibited, it could still be the case that enhanced degradation is occurring by a separate, TRCP-independent pathway that operates in HF-treated cells.
Our response: These experiments are not flawed but a repetition of published data. However, we have complemented our dataset by a different set of experiments. We now also show that treatment with the proteasome inhibitor MG-132 did not rescue the loss ATF4 and R15B observed at high HF concentrations (New Figure 4E) indicating that this does not result from increased degradation. This validates our conclusions with two different inhibitors of protein degradation.

Other points:
-The sentence on p. 8 "R15A induction was not detected in these global proteomic analyses (Figure 3 and Table EV1), although it was manifest on immunoblot analyses (Figure 1A and 1D), most probably because of its low abundance (http://mapofthecell.biochem.mpg.de/) (Itzhak et al., 2016)" ignores the fact that the concentration of HF of 12.5 was too low to induce R15A in Fig. 1A.
Our response: We have deleted the sentence.

-p. 11 and Fig. 5A: they should present the polarity scores for the induced and repressed genes to bolster their conclusions that only the HF-repressed genes show a shift in ribosome density towards the 5' end.
Our response: There are fewer transcripts in the induced and repressed groups than in the global analyses. A polarity score presentation is not adequate for these small groups.

-p. 11: The sentence "The translationally repressed mRNAs displayed an increased ribosome density in their 5' UTR (Figure 5D)" is confusing because the legend seems to indicate that the metagene plots show ribosome occupancies beginning at the start codons of the main coding sequences. As such, they do not report on 5'UTR translation. This is very confusing.
Our response: We don't see a problem with this figure. A dashed line indicates the start site, as indicated in the figure legend. The increased ribosome density is before the start site, in the 5'UTR Figure 5D.

Referee #3:

This is an interesting and well executed study. The authors find that attenuation of ATF4 induction by high doses of halofuginone is caused by translation elongation defects rather than by eIF2alpha kinase regulation. The results are consistent with work cited by the authors showing that ribosome stalling due to low tRNA charging is sufficient to reduce global and gene-specific protein synthesis, and that loss of the GCN2 response to ribosome stalling/uncharged tRNA exacerbates this phenomenon. The authors should consider the following points prior to publication.

1. Figure 4D-F is not particularly convincing. Though MLN4924 clearly affects the baseline level of R15B and Atf4, the proteins are degraded at the same rate or even faster in the presence of MLN4924. Specifically, the slope of the line in 4E is similar or more steep, indicating faster degradation, in the presence of MLN4924. This misinterpretation may be caused by using the 50% level of the protein in the UT condition only to calculate the half life +/- MLN4924, which is misleading because the initial level is so much higher in the presence of the drug. Instead, the 50% level of the protein separately +/- drug should be used to calculate its half life. The real half life of ATF4 +MLN4924 looks to be ~18 minutes, and the real half life of R15B +MLN4924 looks to be ~60 minutes, suggesting that they are degraded similarly in the presence and absence of MLN4924. The data suggest that MLN4924 has little effect on the degradation rate of these proteins, but perhaps increases their expression at baseline. An potentially more convincing way to show that degradation does not explain reduced Atf4/R15B levels at high HF doses, which is important, would be to use a more general proteasome inhibitor. I think it would only be necessary panel F in the presence of a proteasome inhibitor.

We have enjoyed working on the revision. We are pleased to have engaged the reviewer to this level of details and grateful for the insightful comments that contributed to strengthen the study.

Our response: We followed the reviewer's suggestion and performed additional experiments. We now show that treatment with the proteasome inhibitor MG-132 did not rescue the loss ATF4 and R15B observed at high HF concentrations (New Figure 4E) indicating that this does not result from increased degradation.
The experiments showing the effects of MLN4924 on the half-life of R15A and ATF4 are repetition of published data. These are not central to our study and they have therefore been moved to the EV section. The quantifications of these experiments have now been conducted following the reviewer's recommendation.

2. The growth rate data in Figure 7 is difficult to interpret as shown. In any condition where the cells reach 100% confluency after 72 hours, any real growth rate differences before that point are masked because it is unclear when the cells became saturated. For example, one condition could have reached confluency at 48 hours and another at 72 hours, presenting a significant growth rate alteration, and the data would look the same at 72 hours by this measurement. Based on the methods section it seems that the authors have time course data from the IncuCyte that should address this issue. Rather than ,or in addition to, showing % confluency at the end, they should show the full growth curve, and/or calculate the slope from the portion of the curve where cells are in exponential growth.
Our response: We have now added the growth rates for every experiment in the supplementary

data (Figure EV5). In the comparative experiment of the H23 and H441 cell lines, both remain in exponential growth throughout the experiment. Thus, the comparisons of the two cell lines were not affected by saturated cultures. However, inspired by the reviewer's comments, we have presented the slopes of the full growth curves (Figure 7C - 7F) as a robust expression of the recorded differences throughout the experiments, instead of plotting endpoint results normalised to untreated conditions. Additionally, we have plotted the analyses of HF effects in HeLa cells (Figure 7, A-B) at an earlier timepoint of 56h, before any condition within the experiment reaches confluency.

Minor points/suggestions:

1. Can the authors comment on why Atf4/R15A mRNA levels are independent of Atf4 levels?
Our response: The transcriptional control of Atf4 is understudied. Most of the studies focus on the translational control of ATF4 expression. The same comment applies to R15A. The complete mechanism of transcriptional regulation of Atf4 and R15A remains to be elucidated.

2. It may be interesting to correlate the log2 fold changes in figure 3 with the absolute proline content of each protein to further support a ribosome stalling based mechanism for translational repression.
Our response: We have looked into this and found that proline content does not correlate with the decreased abundance of proteins following HF. A potential explanation for this could be the fact that proline is required for translation of most proteins and one proline codon could be enough to stall translation when cells are depleted of prolyl-tRNA.

3. The authors might point out in Figure 5D that increased ribosome density in the coding region of Atf4 could also be caused by slow elongation/ribosome stalling - so it is difficult to interpret this signal, and it does not necessarily indicate increased Atf4 production (in fact it is unclear to me what to expect because 100 nM is around the inflection point for their observed attenuation of Atf4 induction by HF between 62.5 and 200-300 nM). It would be interesting to also show this plot for R15B.
Our response: The increased ribosome density in ATF4 coding region upon HF observed in the ribosome profiling (100 nM HF) coincides with the peak of ATF4 expression. Thus, we think that the interpretation we have provided is the most parsimonious in this case.
We analysed the R15B data but the number of reads for this transcript is too low for the data to be conclusive and therefore we did not present it.

4. The wording of the results description of Figure 4C is confusing - I think the authors mean not statistically significantly different between 4KO and 4KO+GCN2, as they indicate for the description of 4D.
Our response: This has been edited.

5. It may be interesting to speculate that eIF2alpha phosphorylation continues to increase at high HF doses because R15A/B expression are attenuated. However, this could also result from increased activation of GCN2 at high HF doses due to lower tRNA charging and more ribosome
Our response: This is a fair point which has now been added.

Thank you for submitting your revised manuscript. As discussed upon receiving advice on your preliminary response from an additional external expert, we sent the revised manuscript back to referee #3. We have received the report (please see below) and I am pleased to say that s/he now supports publication. Therefore, I would ask you to address a number of editorial issues that are listed in detail below in a final revised version of the manuscript.

Referee #3:

I have no future comments. I am satisfied with the authors revisions.

2nd Revision - Editorial Decision 26th Mar 2022

Thank you again for submitting the final revised version of your manuscript and addressing the remaining points. I am pleased to inform you that we have now accepted it for publication in The EMBO Journal.

EMBO Press Author Checklist

Corresponding Author Name: Anne Bertolotti
Journal Submitted to: EMBO Journal
Manuscript Number: EMBOJ-2021-109985

USEFUL LINKS FOR COMPLETING THIS FORM
The EMBO Journal - Author Guidelines
EMBO Reports - Author Guidelines
Molecular Systems Biology - Author Guidelines
EMBO Molecular Medicine - Author Guidelines

Reporting Checklist for Life Science Articles (updated January 2022)
This checklist is adapted from Materials Design Analysis Reporting (MDAR) Checklist for Authors. MDAR establishes a minimum set of requirements in transparent reporting in the life sciences (see Statement of Task: 10.31222/osf.io/9sm4x). Please follow the journal's guidelines in preparing your manuscript.
Please note that a copy of this checklist will be published alongside your article.

Abridged guidelines for figures
1. Data
The data shown in figures should satisfy the following conditions:
- the data were obtained and processed according to the field's best practice and are presented to reflect the results of the experiments in an accurate and unbiased manner.
- ideally, figure panels should include only measurements that are directly comparable to each other and obtained with the same assay.
- plots include clearly labeled error bars for independent experiments and sample sizes. Unless justified, error bars should not be shown for technical replicates.
- if n<5, the individual data points from each experiment should be plotted. Any statistical test employed should be justified.
- Source Data should be included to report the data underlying figures according to the guidelines set out in the authorship guidelines on Data Presentation.

2. Captions
Each figure caption should contain the following information, for each panel where they are relevant:
- a specification of the experimental system investigated (eg cell line, species name).
- the assay(s) and method(s) used to carry out the reported observations and measurements.
- an explicit mention of the biological and chemical entity(ies) that are being measured.
- an explicit mention of the biological and chemical entity(ies) that are altered/varied/perturbed in a controlled manner.
- the exact sample size (n) for each experimental group/condition, given as a number, not a range;
- a description of the sample collection allowing the reader to understand whether the samples represent technical or biological replicates (including how many animals, litters, cultures, etc.).
- a statement of how many times the experiment shown was independently replicated in the laboratory.
- definitions of statistical methods and measures:
  - common tests, such as t-test (please specify whether paired vs. unpaired), simple χ2 tests, Wilcoxon and Mann-Whitney tests, can be unambiguously identified by name only, but more complex techniques should be described in the methods section;
  - are tests one-sided or two-sided?
  - are there adjustments for multiple comparisons?
  - exact statistical test results, e.g., P values = x but not P values < x;
  - definition of 'center values' as median or average;
  - definition of error bars as s.d. or s.e.m.

Please complete ALL of the questions below.
Select "Not Applicable" only when the requested information is not relevant for your study.

Materials

Newly Created Materials	Information included in the manuscript?	In which section is the information available? (Reagents and Tools Table, Materials and Methods, Figures, Data Availability Section)
New materials and reagents need to be available; do any restrictions apply?	Not Applicable	

Antibodies	Information included in the manuscript?	In which section is the information available? (Reagents and Tools Table, Materials and Methods, Figures, Data Availability Section)
For **antibodies** provide the following information:<br>- Commercial antibodies: RRID (if possible) or supplier name, catalogue number and or/clone number<br>- Non-commercial: RRID or citation	Yes	Materials and Methods

DNA and RNA sequences	Information included in the manuscript?	In which section is the information available? (Reagents and Tools Table, Materials and Methods, Figures, Data Availability Section)
**Short novel DNA or RNA including primers, probes**: provide the sequences.	Yes	Materials and Methods

Cell materials	Information included in the manuscript?	In which section is the information available? (Reagents and Tools Table, Materials and Methods, Figures, Data Availability Section)
**Cell lines:** Provide species information, strain. Provide accession number in repository **OR** supplier name, catalog number, clone number, and/**OR** RRID.	Yes	Materials and Methods
**Primary cultures:** Provide species, strain, sex of origin, genetic modification status.	Not Applicable	
Report if the cell lines were recently **authenticated** (e.g., by STR profiling) and tested for mycoplasma contamination.	Not Applicable	

Experimental animals	Information included in the manuscript?	In which section is the information available? (Reagents and Tools Table, Materials and Methods, Figures, Data Availability Section)
**Laboratory animals or Model organisms:** Provide species, strain, sex, age, genetic modification status. Provide accession number in repository **OR** supplier name, catalog number, clone number, **OR** RRID.	Not Applicable	
**Animal observed in or captured from the field:** Provide species, sex, and age where possible.	Not Applicable	
Please detail **housing and husbandry conditions**.	Not Applicable	

Plants and microbes	Information included in the manuscript?	In which section is the information available? (Reagents and Tools Table, Materials and Methods, Figures, Data Availability Section)
**Plants:** provide species and strain, ecotype and cultivar where relevant, unique accession number if available, and source (including location for collected wild specimens).	Not Applicable	
**Microbes:** provide species and strain, unique accession number if available, and source.	Not Applicable	

Human research participants	Information included in the manuscript?	In which section is the information available? (Reagents and Tools Table, Materials and Methods, Figures, Data Availability Section)
If collected and within the bounds of privacy constraints report on age, sex and gender or ethnicity for all study participants.	Not Applicable	

Core facilities	Information included in the manuscript?	In which section is the information available? (Reagents and Tools Table, Materials and Methods, Figures, Data Availability Section)
If your work benefited from core facilities, was their service mentioned in the acknowledgments section?	Yes	Core facility member listed as an author

Design

Study protocol	Information included in the manuscript?	In which section is the information available? (Reagents and Tools Table, Materials and Methods, Figures, Data Availability Section)
If study protocol has been **pre-registered, provide DOI in the manuscript**. For clinical trials, provide the trial registration number **OR** cite DOI.	Not Applicable	
Report the **clinical trial registration number** (at ClinicalTrials.gov or equivalent), where applicable.	Not Applicable	

Laboratory protocol	Information included in the manuscript?	In which section is the information available? (Reagents and Tools Table, Materials and Methods, Figures, Data Availability Section)
Provide DOI OR other citation details if **external detailed step-by-step protocols** are available.	Not Applicable	

Experimental study design and statistics	Information included in the manuscript?	In which section is the information available? (Reagents and Tools Table, Materials and Methods, Figures, Data Availability Section)
Include a statement about **sample size** estimate even if no statistical methods were used.	Yes	Figures
Were any steps taken to minimize the effects of subjective bias when allocating animals/samples to treatment (e.g. **randomization procedure**)? If yes, have they been described?	Not Applicable	
Include a statement about **blinding** even if no blinding was done.	Not Applicable	
Describe **inclusion/exclusion criteria** if samples or animals were excluded from the analysis. Were the criteria pre-established?<br><br>If sample or data points were omitted from analysis, report if this was due to attrition or intentional exclusion and provide justification.	Not Applicable	No samples were excluded.
For every figure, are **statistical tests** justified as appropriate? Do the data meet the assumptions of the tests (e.g., normal distribution)? Describe any methods used to assess it. Is there an estimate of variation within each group of data? Is the variance similar between the groups that are being statistically compared?	Yes	Figures

Sample definition and in-laboratory replication	Information included in the manuscript?	In which section is the information available? (Reagents and Tools Table, Materials and Methods, Figures, Data Availability Section)
In the figure legends: state number of times the experiment was **replicated** in laboratory.	Yes	Figures
In the figure legends: define whether data describe **technical or biological replicates**.	Yes	Figures

Ethics

Ethics	Information included in the manuscript?	In which section is the information available? (Reagents and Tools Table, Materials and Methods, Figures, Data Availability Section)
Studies involving **human participants**: State details of **authority granting ethics approval** (IRB or equivalent committee(s), provide reference number for approval.	Not Applicable	
Studies involving **human participants**: Include a statement confirming that **informed consent** was obtained from all subjects and that the experiments conformed to the principles set out in the WMA Declaration of Helsinki and the Department of Health and Human Services Belmont Report.	Not Applicable	
Studies involving **human participants:** For publication of **patient photos**, include a statement confirming that consent to publish was obtained.	Not Applicable	
Studies involving experimental **animals**: State details of **authority granting ethics approva**l (IRB or equivalent committee(s), provide reference number for approval. Include a statement of compliance with ethical regulations.	Not Applicable	
Studies involving **specimen and field samples:** State if relevant **permits** obtained, provide details of authority approving study; if none were required, explain why.	Not Applicable	

Dual Use Research of Concern (DURC)	Information included in the manuscript?	In which section is the information available? (Reagents and Tools Table, Materials and Methods, Figures, Data Availability Section)
Could your study fall under dual use research restrictions? Please check biosecurity documents and list of **select agents and toxins** (CDC): https://www.selectagents.gov/sat/list.htm	Not Applicable	
If you used a select agent, is the security level of the lab appropriate and reported in the manuscript?	Not Applicable	
If a study is subject to dual use research of concern regulations, is the name of the **authority granting approval and reference number** for the regulatory approval provided in the manuscript?	Not Applicable	

Reporting

The MDAR framework recommends adoption of discipline-specific guidelines, established and endorsed through community initiatives. Journals have their own policy about requiring specific guidelines and recommendations to complement MDAR.

Adherence to community standards	Information included in the manuscript?	In which section is the information available? (Reagents and Tools Table, Materials and Methods, Figures, Data Availability Section)
State if relevant guidelines or checklists (e.g., **ICMJE, MIBBI, ARRIVE, PRISMA**) have been followed or provided.	Not Applicable	
For **tumor marker prognostic studies,** we recommend that you follow the **REMARK** reporting guidelines (see link list at top right). See author guidelines, under 'Reporting Guidelines'. Please confirm you have followed these guidelines.	Not Applicable	
For **phase II and III randomized controlled trials**, please refer to the **CONSORT** flow diagram (see link list at top right) and submit the CONSORT checklist (see link list at top right) with your submission. See author guidelines, under 'Reporting Guidelines'. Please confirm you have submitted this list.	Not Applicable	

Data Availability

Data availability	Information included in the manuscript?	In which section is the information available? (Reagents and Tools Table, Materials and Methods, Figures, Data Availability Section)
Have **primary datasets** been deposited according to the journal's guidelines (see 'Data Deposition' section) and the respective accession numbers provided in the Data Availability Section?	Yes	Materials and Methods
Were **human clinical and genomic datasets** deposited in a public access-controlled repository in accordance to ethical obligations to the patients and to the applicable consent agreement?	Not Applicable	
Are **computational models** that are central and integral to a study available without restrictions in a machine-readable form? Were the relevant accession numbers or links provided?	Not Applicable	
If publicly available data were reused, provide the respective **data citations in the reference list**.	Yes	Materials and Methods
